# Divergent pathways to depression: a network analysis of adverse childhood experiences in migrant and non-migrant youth

**DOI:** 10.3389/fpsyt.2026.1725710

**Published:** 2026-02-27

**Authors:** Cihang Liu, Xinze Liu, Shujian Wang

**Affiliations:** 1 School of Psychology, Northeast Normal University, Changchun, China; 2 Faculty of Psychology, Beijing Normal University, Beijing, China; 3 Beijing Key Laboratory of Applied Experimental Psychology, National Demonstration Center for Experimental Psychology Education, Beijing, China

**Keywords:** adolescents, adverse childhood experiences, depression, network analysis, rural-to-urban migration

## Abstract

**Background:**

The interplay among Adverse Childhood Experiences (ACEs), rural-to-urban migration, and depression is a critical public health concern, particularly in rapidly urbanizing societies like China. While traditional variable-level studies confirm a general association, they often obscure the granular psychological pathways through which individual traumatic experiences manifest as specific depressive symptoms. Rural-to-urban migration represents a complex environmental transition that may fundamentally reshape these pathways due to unique stressors like social exclusion and identity fragmentation. This study employs a network analysis approach to compare the symptom-level architecture of ACEs and depressive symptoms in migrant and non-migrant university students, identifying key “bridging symptoms” to inform targeted interventions.

**Methods:**

A total of 12,000 university students from Jilin Province, China, completed online questionnaires assessing ACEs and depressive symptoms between September and November 2024. Participants were categorized into rural-to-urban (*N* = 1,491, age = 18.23 ± 1.31) and non-rural-to-urban (*N* = 10,509, age = 18.56 ± 1.52) groups based on their reported residential status during childhood. A symptom network analysis was performed.

**Results:**

12% of children had experienced rural-to-urban migration during childhood, and 53% of adolescents reported exposure to ACEs. In the rural-to-urban group, sexual abuse emerged as the key bridging symptom linking ACEs to depressive symptoms, whereas in the non-rural-to-urban group, household substance abuse was the primary bridging symptom connecting ACEs and depressive symptoms. Moreover, the Network Comparison Test revealed significant differences between sexual abuse and household substance abuse across groups. In addition, sleep problems were identified as a notable depressive symptom within the rural-to-urban group.

**Conclusion:**

This study highlights distinct symptom-level pathways linking ACEs to depressive symptoms in rural-to-urban versus non-rural-to-urban adolescents, emphasizing the pivotal role of sexual abuse among migrants and household substance abuse among non-migrants. Public mental health initiatives targeting adolescents in transitional settings could specifically address the unique risk factors highlighted by these key bridging symptoms.

## 1 Introduction

Rural-to-urban migration among children, driven by parental pursuit of employment, better educational resources, and broader socioeconomic shifts, has become widespread in many countries (1), especially in China (2). In the Chinese context, rural-to-urban migrant children, also referred to as part of the “floating population”, are defined as individuals aged approximately 6 to 15 who move from their registered hukou locality in rural areas to urban centers with their parents and reside there for more than six months (3). By 2020, the population of such children had reached approximately 53.14 million (4), highlighting the magnitude of this demographic shift. A growing body of evidence indicates that rural-urban migrant children are a particularly vulnerable group, disproportionately concentrated in low-income communities characterized by frequent residential turnover, diminished social cohesion, and inadequate public safety (5, 6). These challenging environments sharply increase the risk of adverse childhood experiences (ACEs), which, in migrant populations, may manifest not only as abuse, neglect, or household dysfunction but also as unique migration-linked stressors. Crucially, the cumulative adversities associated with rural-to-urban migration serve not only to elevate ACEs exposure, but also to markedly increase the risk of developing depressive symptoms during adolescence and early adulthood (7, 8).

ACEs are defined as a broad spectrum of harmful experiences occurring during childhood or adolescence, including direct abuse and neglect as well as indirect exposures such as household dysfunction, parental substance abuse or mental illness, family conflict, and separation (9–11). Within the migrant context, children are particularly likely to experience parental separation, unstable caregiving, exposure to substance abuse or mental illness within the family, and elevated rates of social exclusion or bullying in unfamiliar urban schools (12–15). These experiences are shaped by both proximal family dynamics (16) and broader social structural determinants (12). From the perspective of proximal family dynamics, rural-to-urban migration introduces especially pronounced challenges. Migration frequently disrupts family stability, fragments existing social support networks, and places children in unfamiliar, often unwelcoming, environments. As a consequence, the ACEs encountered by migrant children often encompass additional migration-related adversities, including parental absence, unstable caregiving, heightened vulnerability to bullying or social exclusion in new urban settings, and chronic uncertainty regarding housing or schooling (15, 17). Thus, migration not only increases the likelihood of ACEs, but also alters their character and amplifies their psychological impact (18).

On the other hand, from the perspective of broader social structural determinants (12), rural-to-urban migration exposes children to a host of environmental and systemic risks beyond the family. Migrant children frequently encounter institutional barriers, discrimination, and social marginalisation rooted in their rural background or socioeconomic status. These structural challenges often result in restricted access to quality education, limited health resources, and residence in unsafe neighbourhoods, all of which heighten the risk of ACEs such as bullying, exclusion, and community violence. Discriminatory practices and a lack of community cohesion further reinforce feelings of alienation and chronic stress (19, 20). Over time, these external adversities interact with family instability to intensify the frequency of ACEs and the risk of mental health problems (21). It is therefore unsurprising that the cumulative burden of these adversities substantially increases the risk of depression and other mental health problems among migrant children, both in adolescence and early adulthood (7, 18, 22).

From the theoretical perspective of ecological systems theory (23) and cumulative risk theory (24), migration heightens exposure to ACEs and depression risk through multiple, interlocking pathways. Acculturative stress, arising from the necessity to adapt swiftly to new cultural and social environments with limited support, can erode a child’s emotional security and undermine family functioning (25). Discrimination and peer rejection further amplify feelings of exclusion and helplessness, thereby directly increasing the likelihood of both ACEs and depressive symptoms (26). Over time, cumulative exposure to such stressors may result in allostatic load, which can lead to persistent dysregulation of mood and stress response systems, thus increasing vulnerability to depression (27). Consistent with these mechanisms, meta-analytic findings have shown that migrant children exhibit markedly higher rates of mental health symptoms, especially depression, compared to their non-migrant urban peers (28–32). The long-term implications are substantial: childhood exposure to ACEs and maltreatment is robustly linked to an elevated risk of depression persisting into adulthood (30, 33). Moreover, meta-analytic evidence confirms that different forms of ACEs are associated with an increased risk of depression (11, 21, 34), with psychological abuse and neglect demonstrating the strongest associations (35).

Although research on migration, ACEs, and adolescent depressive symptoms has increased, most studies have examined these constructs at an aggregate level and have relied primarily on epidemiological indicators such as correlations and risk ratios (30, 33). These approaches are valuable for estimating population burden and informing public health planning, but they provide limited insight into the symptom level mechanisms through which migration may shape the associations between early adversity and depression. This limitation is important because ACEs are heterogeneous, and different adversities may be linked to different depressive symptoms (36). In addition, these links may vary across developmental and contextual conditions. Migrant youth may face additional psychosocial and environmental demands, including disrupted social ties, school adjustment pressures, and reduced access to local resources, which may strengthen some adversity symptom connections while weakening others (37). Accordingly, a symptom level framework is needed to examine whether migration moderates the pattern of associations between ACEs and depressive symptoms, and to identify pathways that may differ between migrant and non-migrant adolescents. Accordingly, a symptom level network framework is needed to examine whether migrant status moderates the network structure connecting ACEs and depressive symptoms, and to identify the connections that are stronger or weaker across migrant and non-migrant adolescents.

Network analysis offers a novel framework for conceptualizing and analyzing mental disorders by modeling them not as latent constructs, but as systems of symptoms that are directly related to one another. Taking depressive disorder as an example, within the framework of network analysis, it is not “depressive disorder” that causes “depressive symptoms,” but rather “depressive symptoms” that constitute “depressive disorder” (38). This approach enables a more refined analysis of how symptoms interact within a network, with particular emphasis on identifying central symptoms that drive the activation of others and may serve as key targets for intervention (39, 40). While a number of studies have applied network analytic techniques to examine the association between ACEs and diverse mental health problems (41, 42), only a few have explored the relation between depression and constructs closely related to ACEs, such as negative life events (43) and early life adversity (44, 45). In addition, Fengjun et al. (46) showed that central and bridge symptoms differed between groups with and without ACEs in a sample of pregnant women. However, comparable evidence remains limited in adolescents, particularly when ACEs are assessed using structured standard ACE domains rather than broad cumulative scores. Moreover, no study has tested whether the symptom-level associations between specific ACEs and depressive symptoms differ between youth with and without childhood urban migration experience. This gap limits our understanding of migration-related heterogeneity in pathways from adversity to depression. Given the unique constellation of socioeconomic and psychosocial challenges faced by migrant children, this omission represents a substantial gap in understanding how early environmental transitions may shape the internal architecture of depressive symptomatology. By adopting a symptom network-based approach, the present study aims to move beyond traditional categorical models and provide a more nuanced understanding of how complex life transitions shape adolescent mental health.

In summary, rural-to-urban migrant adolescents and their non-migrant counterparts often encounter significantly different family environments, educational settings, and peer relationships. These distinct developmental contexts subject them to different types of ACEs and life stressors, which may in turn manifest as divergent depressive symptom profiles. While previous research has robustly established the general correlation between ACEs and depression, there remains a critical gap in understanding whether the mechanistic process—specifically, how individual traumatic experiences translate into specific depressive symptoms—differs across these two groups. To address this gap, the present study utilizes a symptom-level network framework to explore how childhood migration shapes the internal architecture of adolescent mental health. Specifically, the study pursued two main objectives: (1) to compare the prevalence and severity of ACEs and depressive symptoms between adolescents with and without a history of rural-to-urban migration; and (2) to examine differences in the network structure of ACEs and depressive symptoms across these groups. Children belonging to the floating population are known to face heightened social and environmental pressures, increased family instability, and limited access to educational resources, all of which substantially increase their risk of ACE exposure (13, 14, 17). Consistent evidence also indicates that migrant children are at greater risk of developing depressive symptoms compared to their permanent urban peers (2, 7, 8, 31). Accordingly, the study hypothesizes that (1) adolescents with a migration history will exhibit higher prevalence and severity of both ACEs and depressive symptoms; and (2) their symptom networks will display distinct structural characteristics, including higher overall connectivity and altered centrality and bridging patterns compared to those of adolescents without migration experience.

## 2 Methods

### 2.1 Participants and procedure

Data were collected through an online questionnaire administered at 20 universities in Jilin Province, China between September and November 2024. University staff members distributed the questionnaire link to student chat groups, inviting students to participate. A total of 15,564 individuals accessed the link. Participants who declined to provide consent (N = 332) or failed the attention-check items (N = 3,232) were excluded. After these exclusions, valid responses from 12,000 participants were retained for subsequent analyses.

To address the aims of the current study, participants were classified based on their residential status between the ages of 6 and 15, using the following question: “During the ages of 6 to 15, what was your living situation?” The response options were: (1) always lived in a rural area, (2) always lived in an urban area, and (3) moved from a rural to an urban area (for more than six months). Participants who selected option (3) were categorized into the rural-to-urban group, while those who selected either option (1) or (2) were categorized into the non-rural-to-urban group. Of the 12,000 participants, 1,491 were assigned to the rural-to-urban group, while 10,509 comprised the non-rural-to-urban group. Within the rural-to-urban group, 942 were male, with a mean age of 18.23 years (*SD* = 1.31). In the non-rural-to-urban group, 2,239 were male, with a mean age of 18.56 years (*SD* = 1.52). Regarding household composition, 8,898 participants (74.15%) were only children. In terms of overall residency, 9,722 students (81.02%) lived in rural areas, while 2,278 (18.98%) lived in urban areas. 6,483 students (54.03%) were enrolled in undergraduate programs, and 5,517 (45.98%) were enrolled in associate degree programs.

All participants were informed of the study’s purpose before completing the questionnaire. This study was approved by the Academic Ethics Committee of the School of Psychology, Northeast Normal University (IRB number: 2022038).

### 2.2 Measures

#### 2.2.1 Adverse childhood experiences international questionnaire

The ACE-IQ (47), which has been validated in the Chinese population (48), was used to assess individuals’ exposure to ACEs during the first 18 years of their lives. The ACE-IQ comprises 13 categories of adverse experiences, assessed through a total of 29 items: emotional abuse (2 items); physical abuse (2 items); contact sexual abuse (4 items); alcohol and/or drug abuser in the household (1 item); someone chronically depressed, mentally ill, institutionalized or suicidal (1 item); incarcerated household member (1 item); one or no parents, parental separation or divorce (2 items); household member treated violently (3 items); emotional neglect (2 items); physical neglect (3 items); bullying (1 item); community violence (3 items); and collective violence (4 items). Note that, in the context of China, collective violence was excluded from the analysis due to the absence of war or related collective violence incidents.

ACE-IQ has two scoring methods, binary and frequency versions. In the binary version, any affirmative experience (even once) scores 1 point in the respective category, and in the frequency version, only experiences meeting specific recurrence criteria (e.g., “many times”) are counted. The overall ACE score ranges from 0 to 12 in the current study, with higher scores indicating greater ACEs. The present study used the binary scoring method to provide a more conservative and internationally comparable estimate of ACE exposure. The recommended cut-off score for ACE-IQ to be 4 (49). The ACE-IQ demonstrated good internal consistency in the current sample, with a Cronbach’s *α* coefficient of 0.80.

#### 2.2.2 Center for epidemiologic studies depression scale-20 item version

The CESD-20 is a widely used self-report questionnaire designed to assess depressive symptoms in the general population (50). This scale comprises 20 items that evaluate various aspects of depression. The symptoms addressed include depressed mood, feelings of sadness, guilt, hopelessness, helplessness, as well as sleep disturbances, appetite changes, and mental/motor slowness. Each item is rated on a 4-point Likert scale ranging from 0 (“rarely or none of the time”) to 3 (“most or all of the time”), based on the frequency of symptoms experienced during the past week. While a high total score does not confirm a clinical diagnosis of depression, it may indicate the need for further psychological evaluation (51). Following established benchmarks, cut-off scores of 16 and 21 were employed to identify mild-to-moderate and severe depressive symptoms, respectively, with the latter indicating a potential risk for major depressive disorder (52, 53). The CESD-20 demonstrated good cross-cultural reliability and validity (54). In this study, internal consistency was high (Cronbach’s *α* = 0.85).

#### 2.2.3 Demographic information

In addition to the primary scales, participants provided demographic information via the self-report questionnaire, including gender, age, current residence, and education level. These variables were collected to provide a comprehensive profile of the study population and to facilitate subsequent sensitivity evaluations.

### 2.3 Statistical analysis

All analyses were conducted using R software (version 4.3.2). Descriptive analyses were first performed to summarize demographic characteristics (i.e., age and migrant status) and study variables across the total sample. Subsequently, network analyses were conducted to examine the associations between depressive symptoms and ACEs.

Network modeling and visualization were carried out using the bootnet and qgraph packages in R (39, 55). Since the current data are mixed (CESD scores for continuous variables and ACE scores for discrete variables), we employed the mgm method to model the mixed data network structures (56). In the graphical visualization, each node corresponds to a depressive or ACE symptom, while edges reflect the statistical associations between them. Positive and negative associations are indicated by blue and red edges, respectively, with edge thickness signifying the strength of the relationship (57).

To identify key symptoms within the networks, we calculated both strength and bridge strength centrality for each node using the centralityPlot function (58). Strength quantifies a node’s overall influence within the network by summing the weights of all its direct connections. In contrast, bridge strength measures the strength of a node’s connections to nodes in other communities, thereby indicating its bridging function across distinct symptom clusters (59, 60).

The accuracy and stability of the estimated networks were systematically examined using the R package bootnet 1.4.3 (57). To begin with, we employed a nonparametric bootstrapping test to assess 95% confidence intervals (*CI*s) for edge weight. Narrow *CI*s were taken to indicate a reliable edge ranking. A case-dropping bootstrap procedure was implemented to assess the stability of centrality metrics, yielding the correlation stability coefficient (CS-C). This coefficient estimates the largest portion of data that can be excluded while still retaining, with 95% probability, a minimum correlation of 0.70 between the centrality scores of the full and reduced networks. According to widely accepted guidelines, CS-C values exceeding 0.25, 0.50, and 0.75 correspond to acceptable, good, and excellent levels of stability, respectively (61). In addition, we conducted bootstrapped difference tests to evaluate whether variations in edge weights and centrality indices across nodes reached statistical significance.

To examine group-level differences associated with childhood residential mobility, network comparisons were conducted using the NetworkComparisonTest (NCT) package in R (62). This method provides four core statistical tests between rural-to-urban and non-rural-to-urban group: the network structure invariance test assesses whether the overall arrangement of connections differs between groups; the global strength invariance test compares the differences in the total edge strengths across groups; the edge strength invariance test focuses on localized differences in individual edge weights; and the centrality invariance test determines whether node centralities vary significantly between groups.

Finally, we conducted sensitivity analyses to address potential biases stemming from unequal sample sizes and gender imbalances between the groups. To address the former, we followed a subsampling procedure (63) in which we repeatedly drew random subsamples from the non-rural-to-urban group to match the size of the rural-to-urban group. We estimated network parameters for each subsample and calculated the differences between these parameters and those of the original non-rural-to-urban network. Following 1,000 iterations, we generated 95% confidence intervals (CIs) for these differences; CIs encompassing zero indicated that the results were robust to sample size imbalances.

We also examined the potential influence of gender on the study’s findings. Previous research has yielded inconsistent conclusions regarding gender differences in how early adversity impacts adult depressive symptoms. For instance, Gallo et al. (64) reported no statistically significant gender differences in the association between physical or sexual abuse and adult depression. Similarly, Hovens et al. (65) found that adjusting for gender did not significantly alter the relationship between childhood trauma and the course of adult depressive disorders. Conversely, Wei et al. (66) observed that the association between ACEs and depressive symptoms was stronger in females than in males. Furthermore, the interaction between resilience and emotional abuse regarding depressive symptoms appears to differ by gender, a finding supported by Monteiro et al. (67). Given the gender imbalance between the migrant and non-migrant groups in the present study, evaluating the influence of gender on our results was essential. Following the approach of Niu et al. (68), we estimated new network structures that included gender as a covariate. We then calculated correlation coefficients between the adjacency matrices of the original networks and the gender-controlled networks. If the test results showed a very high correlation, it indicated that the primary findings were robust to the influence of gender.

## 3 Results

### 3.1 Demographic information

Demographic information is shown in **Table 1**. The prevalence of mild-to-moderate depressive symptoms (CESD ≥ 16) was 49.4% for the rural-to-urban group and 84.3% for the non-rural-to-urban group. Furthermore, the prevalence of severe depressive symptoms (CESD ≥ 21) was 34.3% and 75.5% for the respective groups. The prevalence of depressive symptoms was significantly higher in the non-rural-to-urban group than in the rural-to-urban group, *χ*²(1) = 984.61, *p* <.001. Similarly, ACEs were more prevalent in the non-rural-to-urban group, with 59.0% reporting at least one ACE, compared to only 7.7% in the rural-to-urban group, *χ*²(1) = 1374.91, *p* < 0.001.

**Table 1:** Demographic information.

Demographic variables	Migration (N = 1491)	Non-migration (N = 10509)	χ^2^	*p*
Age	18.23 (1.31)	18.56 (1.52)		
Gender			1173.10	<0.001
Male	942 (63.2%)	2239 (21.3%)		
Female	549 (36.8%)	8270 (78.7%)		
Only child			374.31	<0.001
No	692 (46.4%)	2410 (22.9%)		
Yes	799 (53.6%)	8099 (77.1%)		
Residence			176.86	<0.001
Urban	472 (31.7%)	1806 (17.2%)		
Rural	1019 (68.3%)	8703 (82.8%)		
Education level			1711.40	<0.001
Bachelor	60 (4.02%)	6423 (61.1%)		
Associate	1431 (96.0%)	4086 (38.9%)		
Depressive symptoms			984.61	<0.001
No	754 (50.6%)	1655 (15.7%)		
Yes	737 (49.4%)	8854 (84.3%)		
ACEs			1374.91	<0.001
No	1376 (92.3%)	4310 (41.0%)		
Yes	115 (7.71%)	6199 (59.0%)		

### 3.2 Descriptive information

Means, standard deviations, skewness, kurtosis, and t-test results for the two groups are presented in **Table 2**. In comparing the item-level means between the rural-to-urban and non-rural-to-urban groups, results revealed significant group differences across all CESD and ACE items. The non-rural-to-urban group scored higher than the rural-to-urban group on most depressive symptoms and ACE exposure levels.

**Table 2:** Item descriptive information and t-test results of the two groups.

Items	Label	Migration (n = 1491)				Non-migration (n = 10509)						Cohen’s *d*
		*M*	*SD*	Skew	Kurtosis		*M*	*SD*	Skew	Kurtosis	*p*	
CESD1	Irritability	1.56	1.00	0.57	-1.21		2.44	0.96	-1.21	-0.26	<0.001	-0.91
CESD2	Poor Appetite	1.10	0.92	1.03	0.33		2.18	1.14	-0.80	-1.08	<0.001	-0.97
CESD3	Helplessness	1.09	0.99	0.92	-0.15		2.19	1.16	-0.86	-1.00	<0.001	-0.96
CESD4	Self Worth	0.47	0.91	1.54	0.69		1.38	0.95	-0.71	-1.32	<0.001	-0.97
CESD5	Concentration Trouble	1.35	0.96	0.83	-0.50		1.16	0.71	1.69	2.66	<0.001	0.25
CESD6	Depressed Mood	1.19	0.91	1.01	0.19		2.22	1.10	-0.82	-1.02	<0.001	-0.96
CESD7	Fatigue	0.92	1.02	1.07	0.05		2.23	1.12	-0.89	-0.92	<0.001	-1.18
CESD8	Hopelessness	0.52	0.94	1.38	0.22		0.29	0.75	2.31	3.77	<0.001	0.29
CESD9	Sense of Failure	0.78	0.99	1.29	0.62		2.14	1.19	-0.78	-1.14	<0.001	-1.17
CESD10	Fear	0.83	1.02	1.19	0.30		2.15	1.17	-0.77	-1.13	<0.001	-1.14
CESD11	Sleep Problems	0.92	1.08	1.03	-0.28		1.05	0.69	1.55	3.34	<0.001	-0.17
CESD12	Happiness	0.35	0.80	1.95	2.17		0.26	0.74	2.68	5.70	<0.001	0.12
CESD13	Reduced Speech	1.19	0.94	0.94	-0.03		2.18	1.16	-0.82	-1.05	<0.001	-0.87
CESD14	Loneliness	1.10	0.94	1.00	0.22		2.17	1.16	-0.80	-1.08	<0.001	-0.94
CESD15	Rejection Feeling	0.73	0.96	1.37	0.95		0.95	0.61	1.47	4.95	<0.001	-0.33
CESD16	Life Satisfaction	0.40	0.86	1.80	1.60		0.29	0.78	2.49	4.64	<0.001	0.14
CESD17	Crying	1.25	0.91	1.01	0.10		1.10	0.65	1.87	4.21	<0.001	0.21
CESD18	Sadness	1.02	1.08	0.89	-0.50		2.21	1.13	-0.86	-0.97	<0.001	-1.05
CESD19	Feeling Disliked	0.77	0.99	1.30	0.63		0.94	0.63	1.41	4.38	<0.001	-0.25
CESD20	Hopelessness	0.64	0.92	1.55	1.55		2.05	1.24	-0.66	-1.34	<0.001	-1.16
ACE1	Emotional Neglect	0.10	0.29	2.74	5.52		0.06	0.25	3.56	10.66	<0.001	0.13
ACE2	Physical Neglect	0.10	0.31	2.59	4.73		0.62	0.49	-0.50	-1.75	<0.001	-1.11
ACE3	Substance Abuse Household	0.04	0.20	4.47	17.96		0.58	0.49	-0.32	-1.90	<0.001	-1.14
ACE4	Mental Illness Household	0.04	0.21	4.39	17.27		0.02	0.14	6.74	43.44	<0.001	0.16
ACE5	Incarcerated Member	0.04	0.19	5.01	23.13		0.02	0.14	7.11	48.63	<0.001	0.12
ACE6	Parental Separation	0.17	0.37	1.78	1.16		0.63	0.48	-0.53	-1.72	<0.001	-0.98
ACE7	Domestic Violence	0.33	0.47	0.71	-1.50		0.61	0.49	-0.47	-1.78	<0.001	-0.58
ACE8	Emotional Abuse	0.06	0.25	3.55	10.58		0.02	0.14	6.74	43.44	<0.001	0.28
ACE9	Physical Abuse	0.05	0.23	3.90	13.22		0.02	0.14	7.10	48.35	<0.001	0.24
ACE10	Sexual Abuse	0.30	0.46	0.88	-1.23		0.61	0.49	-0.45	-1.80	<0.001	-0.64
ACE11	Bullying	0.03	0.18	5.07	23.67		0.01	0.11	9.00	79.07	<0.001	0.19
ACE12	Community Violence	0.07	0.25	3.42	9.67		0.02	0.15	6.26	37.22	<0.001	0.27

### 3.3 Network structures

**Figure 1:**
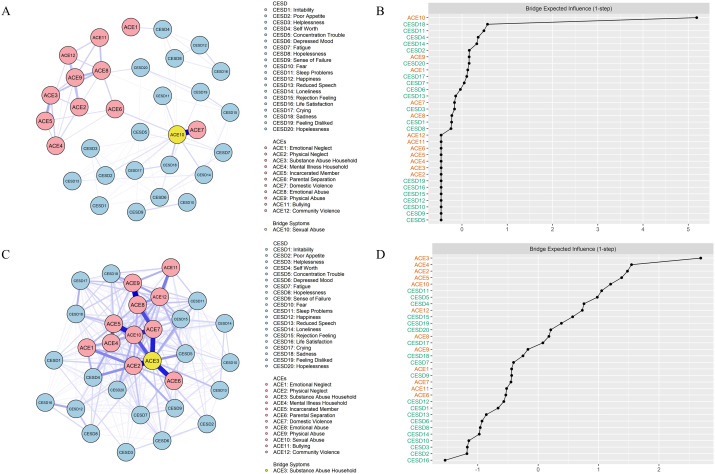
Network structures and centrality indices. Blue lines represent positive associations between symptoms, while red lines indicate negative associations. The thickness of an edge corresponds to the strength of the association. The key channel variables are labeled in yellow. **(A)**, network structure for the rural-to-urban group. **(B)**, standardized bridge EI values among rural-to-urban migrant adolescents. **(C)**, network structure for the non-rural-to-urban group. **(D)**, standardized bridge EI values among non-rural-to-urban migrant adolescents.

**Figures 1A, B**, illustrate CESD-ACE networks for rural-to-urban and non-rural-to-urban groups, while **Figures 1C, D** provide information on bridge EI values for all items. The original adjacency matrices, encompassing all edge weights, can be found in **Supplementary Tables S1**, **S2** in the **Supplementary Materials**. All nodes’ EI values are provided in **Supplementary Figure S1**.

For students with migration experiences, the central symptom was #ACE10 (“Sexual Abuse”), with bootstrapped difference tests indicating higher EI value than most other symptoms (refer to **Supplementary Figure S2**). The symptom with the highest bridge EI value was also #ACE10 (“Sexual Abuse”), with bootstrapped difference tests indicating higher bridge EI value than most other symptoms (refer to **Supplementary Figure S3**). Such results indicate that sexual abuse serves as the channel between depressive symptoms and ACEs.

For students without migration experiences, the central symptom was #ACE3 (“Substance Abuse Household”), with bootstrapped difference tests indicating higher EI value than most other symptoms (refer to **Supplementary Figure S2**). The symptom with the highest bridge EI value was also #ACE3 (“Substance Abuse Household”), with bootstrapped difference tests indicating higher bridge EI value than most other symptoms (refer to **Supplementary Figure S3**).

### 3.4 Network comparison test

**Figure 2:**
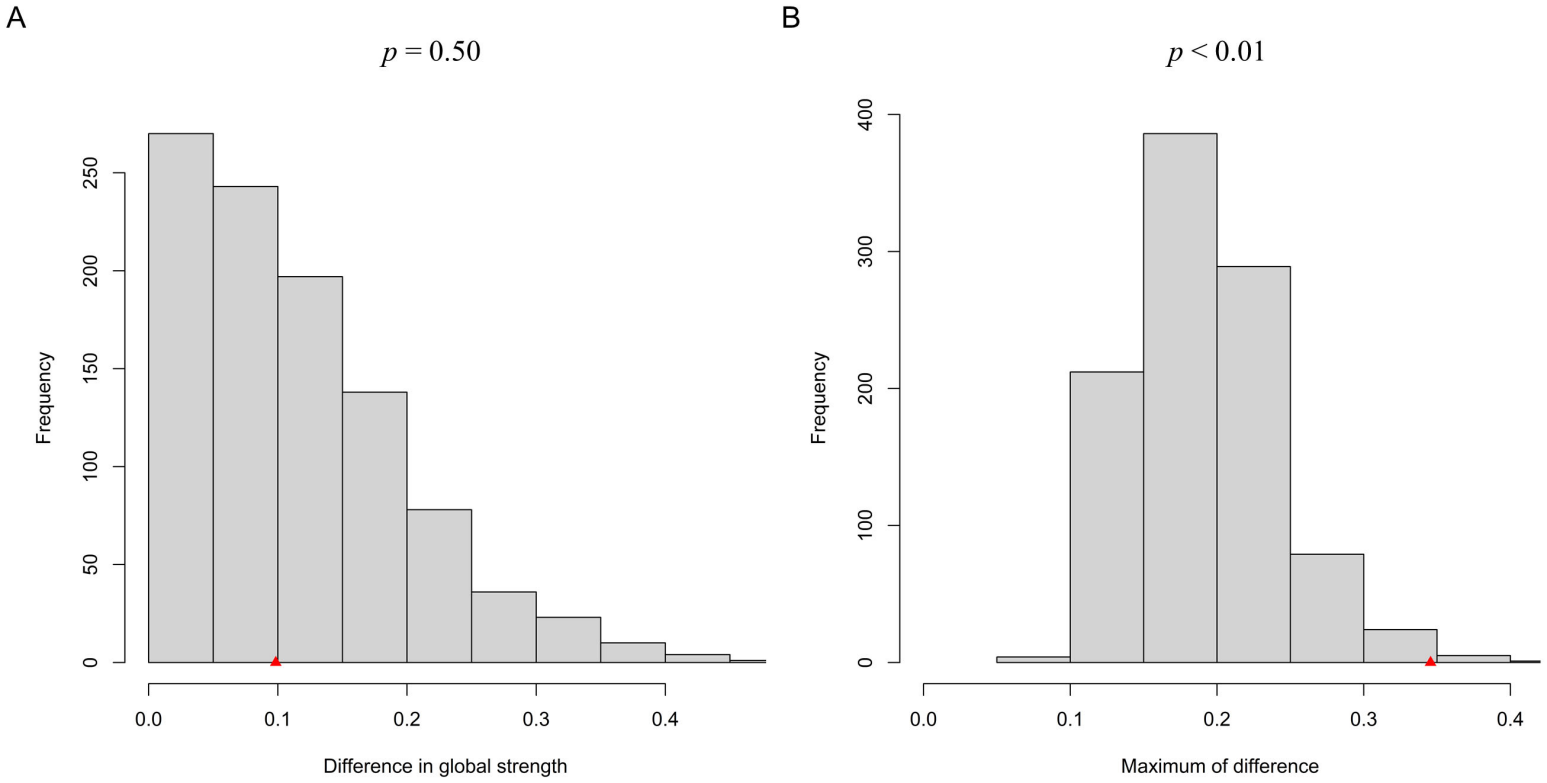
Network comparison test results between rural-to-urban and non-rural-to-urban migrant adolescents. **(A)**, network global invariance test. **(B)**, network structure invariance test.

A permutation test evaluated network edge and global invariance between migration and non-rural-to-urban groups (see **Figure 2**). The global invariance test revealed no significant difference in network global strength between the two groups (*p* = 0.50). However, the edge invariance test indicated a significant difference in edge distribution (*p* < 0.01). Specifically, the bridge EI value of ACE3 (“Substance Abuse Household”) in the non-rural-to-urban group was significantly higher than that in the rural-to-urban group (bEI_non-migration_ = 3.91, bEI_migration_ = 0.00, *p* < 0.05). The bridge EI value of ACE10 (“Sexual Abuse”) in the rural-to-urban group was significantly higher than that in the non-rural-to-urban group (bEI_non-migration_ = 2.59, bEI_migration_ = 2.63, *p* < 0.001). Such results indicate that the transmission mechanisms between depressive symptoms and ACEs are significantly different between migration and non-rural-to-urban groups.

### 3.5 Network accuracy and stability

**Figure 3:**
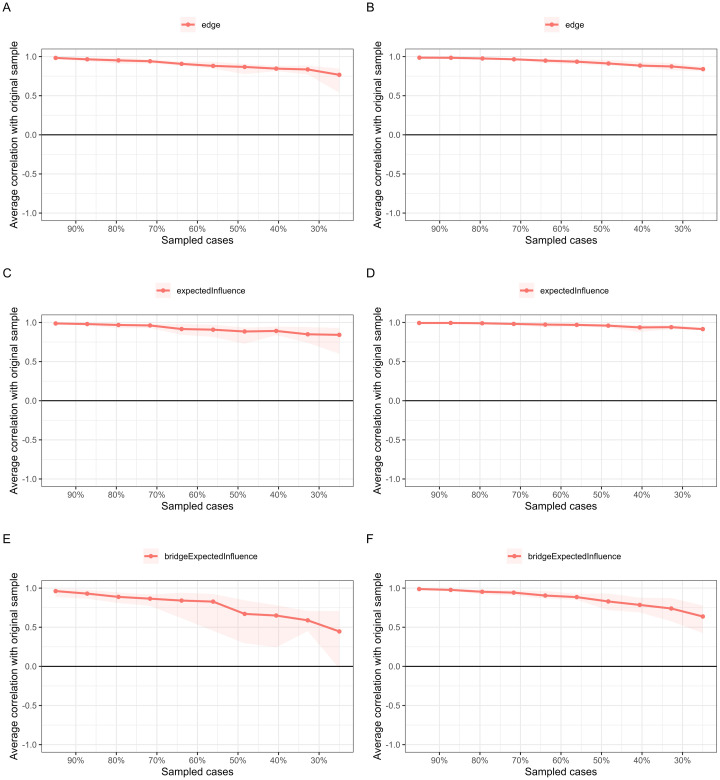
Case-dropping bootstrap test of edge and centrality indices. The x-axis indicates the percentage of cases of the original sample included at each step. The y-axis indicates the correlations between the centrality indices from the original network and the indices from the networks re-estimated after excluding increasing percentages of cases. **(A)**, case-dropping results of edges for the rural-to-urban group. **(B)**, case-dropping results of edges for the non-rural-to-urban group. **(C)**, case-dropping results of EI for the rural-to-urban group. **(D)**, case-dropping results of EI for the non-rural-to-urban group. **(E)**, case-dropping results of bridge EI for the rural-to-urban group. **(F)**, case-dropping results of bridge EI for the non-rural-to-urban group.

The case-dropping analysis results are presented in **Figure 3**. CS-Cs for EI were 0.59 for the rural-to-urban group and 0.75 for the non-migration. The CS-Cs for edges in two groups were 0.67 and 0.75, while the CS-Cs for bridge EI were 0.36 and 0.59, respectively. Such results indicate that current findings are robust. Edge confidence interval plots displayed small to moderate confidence intervals around edge weights, suggesting accurate networks (refer to **Supplementary Figure S3**). Furthermore, the main edges contributing to conclusions were significantly stronger than most other edges (refer to **Supplementary Figure S4**).

### 3.6 Sensitivity analysis

Regarding the sample size imbalance, the subsampling analysis revealed that the 95% CIs for all differences between the original parameters and the small sample parameters contained zero, indicating that sample size did not significantly influence the network structure. The full results are presented in the **Supplementary Table S3**.

Concerning the gender imbalance, the gender-controlled network for the rural-to-urban group was nearly identical to the original network (r = 0.99, p < 0.001). Similarly, the gender-controlled network for the non-rural-to-urban group mirrored its original counterpart (r = 0.99, p < 0.001). These results suggest that the overall network structure remained robust and stable regardless of gender.

### 3.7 The exploratory analysis of childhood residential areas

We conducted exploratory analyses to determine whether childhood residential environments—specifically rural versus urban settings—influenced the ACE-depression network architecture. Among participants without migration history, 8,544 resided in rural areas during childhood (ages 6–15), while 1,965 resided in urban areas (see **Supplementary Figure S6** for network visualizations). For the urban group, sexual abuse (#ACE10) emerged as the most central symptom, while emotional neglect (#ACE1) and low self-worth (#CESD4) functioned as the primary bridge symptoms. Conversely, in the rural group, household substance abuse (#ACE3) was identified as both the most central and the primary bridge symptom, consistent with the findings for the broader non-migrant population. Detailed EI and bridge EI values are provided in **Supplementary Figures S7**, **S8**.

The NCT results indicated that network density was significantly higher in the urban group than in the rural group (p < 0.05). Although the edge invariance test revealed significant differences in the distribution of edge weights (p < 0.05), the strength invariance test showed no significant differences in the centrality indices of any individual nodes. These findings suggest that while the magnitude of specific associations may vary, the overall patterns of connectivity between ACEs and depressive symptoms remain relatively consistent across urban and rural childhood environments. Detailed NCT results are presented in **Supplementary Figure S9**.

## 4 Discussion

Leveraging a large-scale dataset of Chinese adolescents, the present study directly addressed the two pre-specified objectives by exploring how rural-to-urban migration influences the prevalence, severity, and symptom-level network structure of ACEs and depressive symptoms. The network analysis findings partially supported the proposed hypotheses and yielded novel insights that warrant further discussion.

The first noteworthy finding of this study was that 12% of adolescents had experienced rural-to-urban migration during childhood, and 53% had reported exposure to at least one ACE. These prevalence rates are broadly consistent with prior population-based data, such as the survey by Mao and Zhao (69), which reported that 20.8% of migrants had experienced migration before age 14. In terms of ACE exposure, our findings align with those of Cheng et al. (5), who found that children from migrant families frequently encounter adverse events, including trauma, domestic violence, and other forms of household dysfunction. Together, these results demonstrate the substantial proportion of adolescents in China affected by both migration and early-life adversity, highlighting the importance of investigating their joint impact on mental health outcomes.

The second noteworthy phenomenon is the remarkably high prevalence of depressive symptoms identified in this study (34.3% for the rural-to-urban group and 75.5% for the non-migrant group). While the proportion for the migrant group aligns with previous findings (70–73), the 75.5% rate observed in the non-migrant group deviates markedly from established norms. Two potential factors may explain this disparity. First, manageable exposure to early-life challenges may have fostered psychological resilience in the rural-to-urban group. Recent research suggests that individuals with childhood migration experiences demonstrate greater resilience and superior mental health outcomes in adulthood compared to those without such experiences (74). Second, the unexpectedly high prevalence in the non-migrant group may be interpreted through the Prevalence Inflation Hypothesis (75). This hypothesis posits that mental health awareness campaigns, while beneficial, can inadvertently encourage the over-interpretation of mild or transient distress as clinical symptoms. In the Chinese context, non-migrant students typically receive earlier and more systematic mental health education than their rural-to-urban counterparts. While this exposure enhances mental health literacy, it may also lower the threshold for labeling normative stress as “depressive symptoms” on self-report measures like the CES-D. Consequently, the non-migrant group may be more prone to pathologizing routine emotional distress, whereas rural-to-urban students—having had less exposure to psychological discourse—may adopt a more conservative reporting style, only endorsing items when symptoms are severe.

Furthermore, we observed that the proportion of males in the rural-to-urban group was significantly higher than that of females, a finding consistent with previous research (76, 77). Duan et al. (76) argued that due to China’s household registration barriers and the high cost of urban living, city migration is treated as a scarce and costly family resource. Driven by traditional patriarchal values and societal expectations favoring sons, rural families often prioritize male descendants’ access to urban educational resources and cultural capital, anticipating their upward mobility in the future labor market. Tan discussed this phenomenon through the lens of the Split Labor Reproduction Model (SLRM, 78). SLRM was proposed by Burawoy (79). In a stable economic system, wages should ideally support a worker’s entire family, thereby ensuring the total reproduction of labor. However, in rapidly urbanizing societies, migrant workers’ wages are often insufficient to sustain urban livelihoods for a full family unit. Consequently, labor force reproduction is split into two distinct components: the maintenance component, encompassing the immediate physiological expenditures (e.g., food, housing, and transportation) required for daily labor, which occurs within urban areas; (2) the renewal component, the long-term regeneration of the labor force, including childcare, eldercare, and retirement provisions, which is pushed back to rural areas. In the context of rural China, girls often bear the burden of household responsibilities and eldercare (80, 81). As a result, daughters are often “left behind” in rural regions as substitute labor to fulfill the renewal requirements of labor reproduction, while sons are granted the opportunity for urban migration.

Exploratory analysis revealed that while the ACE-depressive symptom association patterns did not differ significantly between participants with urban versus rural childhood residences, the global strength (density) of these associations was significantly higher in the urban group. This finding aligns with existing literature. For instance, a systematic review by Kohler and Penkalla (82) indicated that the prevalence of mood, anxiety, and substance use disorders is consistently higher in urban environments. Neurobiological evidence further elucidates this disparity. Lederbogen et al. (83) demonstrated that individuals raised in urban settings exhibit heightened amygdala sensitivity—the region central to threat detection and fear responses—when subjected to stress. Simultaneously, these individuals show diminished activity in the perigenual anterior cingulate cortex, a region critical for the top-down regulation of amygdala stress responses and negative affect. This neurobiological profile suggests that urban-raised children may be more susceptible to severe depressive reactions following ACEs due to a combination of heightened biological sensitivity to stressors and impaired regulatory mechanisms. Furthermore, from an environmental psychology perspective, Kaplan (84) posits that natural environments facilitate psychological regulation and attention restoration. Children in urban settings may lack access to these “natural pressure relief valves,” potentially allowing early trauma to coalesce into more persistent depressive symptoms without the buffering effects of restorative environments.

Another interesting finding of the current study was that, contrary to our first hypothesis, adolescents in the non-rural-to-urban group reported higher levels of certain ACEs and depressive symptoms compared to their rural-to-urban counterparts. One plausible explanation lies in the imbalance in group sizes, as the majority of participants had no migration experience and, within this subgroup, most had spent their childhood in rural settings. This partially aligns with prior evidence suggesting that adolescents residing in rural areas may be more vulnerable to psychological difficulties due to limited access to mental health resources, lower socioeconomic status, and reduced social support (85, 86). From the perspective of the Stress-Vulnerability Model (87), the chronic environmental stressors characteristic of rural contexts, such as persistent economic strain and fewer educational and psychosocial resources, may interact with individual vulnerability factors to heighten the risk of both ACE exposure and depressive symptom development. Although unexpected, this finding emphasizes the presence of meaningful differences in depressive and ACE-related symptomatology between adolescents with and without migration experience, reinforcing the need for tailored intervention strategies (88) that address both residential context and cumulative life stressors.

From a symptom-level perspective, and in line with our second hypothesis regarding network differences, the present analysis revealed that the identity of bridging symptoms differed substantially between groups, with sexual abuse bridging ACEs and depressive symptoms in the rural-to-urban migrant group, and household substance abuse serving this role in the non-rural-to-urban group. These group-specific bridging patterns are consistent with the Network Theory of Psychopathology (38), which emphasizes that the configuration of symptom-to-symptom connections can differ across subpopulations in ways that meaningfully influence intervention targets. In the ACE framework, sexual abuse refers to any sexual act or interaction between an adult or an older child and a minor undertaken for sexual gratification or financial gain, including but not limited to sexual touching, statutory rape, molestation, prostitution, indecent exposure, involvement in pornography, incest, and other forms of sexual exploitation (47). The observed bridging effect of sexual abuse is consistent with extensive evidence demonstrating a robust association between childhood sexual abuse and later depressive symptoms (89, 90) and suggests that this form of trauma may represent a critical pathway through which early adversity activates and sustains depressive symptomatology.

In addition, among non-rural-to-urban migrant adolescents, household substance abuse was found to be the central bridge symptom linking ACEs and depressive symptoms. In the field of ACEs, household substance abuse refers to the harmful use or dependence on alcohol or illicit drugs by a parent or other household member, which can undermine caregiving, reduce emotional availability, and destabilize the family environment (11, 13, 49). In the Chinese context, this construct primarily reflects parental alcohol misuse, as illicit drug use among parents is relatively rare due to stringent legal regulation and cultural norms (15, 31). The Family Systems Theory (91) emphasizes that persistent parental alcohol misuse can disrupt family boundaries, erode role clarity, and impair communication patterns, creating a relational environment characterized by neglect, emotional unavailability, and inconsistent discipline. Within a network analytic framework (38), such chronic dysfunction may act as a bridge transmitting the effects of other ACEs into depressive symptomatology, activating key cognitive–affective symptoms such as hopelessness, worthlessness, and anhedonia (7, 35).

The prominence of household substance abuse as a bridging symptom in non-migrant adolescents may be further explained by the Stress-Vulnerability Model (87). In relatively stable residential contexts, the stressors associated with parental alcohol misuse are often chronic rather than acute, leading to prolonged exposure that gradually erodes adolescents’ coping capacity and emotional regulatory resources. Over time, this sustained stress can heighten vulnerability to depressive symptoms by altering stress-response systems and reinforcing maladaptive interpersonal schemas. In contrast, rural-to-urban migrant adolescents may experience a broader set of acute migration-linked stressors, such as parental absence, unstable caregiving, and institutional exclusion, which may overshadow the role of household substance abuse in their symptom networks, resulting in different bridge symptom configurations.

From a prevention and intervention perspective, these findings underscore the importance of embedding alcohol-specific, family-focused interventions within school and community health services for non-migrant populations. Early ACE screening in educational and primary care settings (47), coupled with evidence-based parental alcohol misuse programs (92), could disrupt this critical bridge pathway and reduce the propagation of depressive symptoms within the network. Targeting household substance abuse may not only alleviate the direct harms of alcohol misuse but also weaken the indirect transmission of risk from other ACEs to depressive symptoms, thereby producing a cascading reduction in overall mental health burden (38, 42).

### 4.1 Implications

Some implications based on the current results warrant discussion. The identification of sexual abuse as the principal bridging symptom between ACEs and depressive symptoms in rural-to-urban migrant adolescents, and household substance abuse in non-rural-to-urban migrant adolescents, highlights the need for differentiated intervention strategies. For migrant adolescents, multi-tiered, evidence-informed trauma-responsive school interventions (88), which integrate staff training on sexual abuse recognition and response, the establishment of safe and supportive learning environments (93), culturally responsive pedagogical practices (94), and mental health services (95), are essential to mitigate trauma-related sequelae and prevent retraumatization. For non-rural-to-urban migrant adolescents, school-based support groups (96) and family-centered engagement with addiction treatment services (92) should be adapted to address the psychosocial and emotional consequences of household substance misuse. From a prevention perspective, embedding ACE screening within routine school health assessments and primary care visits may facilitate earlier detection across both groups. Policy measures that ensure equitable access to culturally and contextually adapted mental health services, particularly in urban districts with high concentrations of rural-to-urban internal migrants, are critical.

### 4.2 Limitations

Although the present study draws strength from its large, population-based sample of Chinese adolescents and the application of sensitivity analyses that confirmed the robustness of the main findings, several limitations should be acknowledged. First, the retrospective nature of the data collection may have introduced recall bias (97, 98) in participants’ self-reports of ACEs, which could limit the generalizability of the findings. Second, despite the overall large sample size, there was a substantial imbalance in the sizes of the rural-to-urban and non-rural-to-urban groups. While sensitivity analyses indicated that this disparity did not materially affect the results, future research would benefit from more balanced sampling to enhance the robustness of between-group comparisons. Third, the cross-sectional design precludes causal inferences regarding the temporal or directional relationships between ACEs and depressive symptoms; longitudinal designs are needed to clarify the stability and causality of the observed network structures. Finally, all variables were assessed through self-report questionnaires, which may be susceptible to social desirability bias and underreporting of stigmatized experiences such as sexual abuse or household substance misuse, potentially leading to conservative estimates of prevalence and network associations. Furthermore, the current study focused primarily on distal factors (i.e., ACEs) and did not account for the potential influence of recent adverse life events. Proximal stressors prevalent among university students—such as excessive academic pressure, exam-related anxiety, and recent interpersonal conflicts—are well-documented triggers for depressive symptoms (99–101). These contemporary events may function as “matching stressors” that activate latent vulnerabilities established by early childhood adversity. Consequently, the absence of these data precludes a clear differentiation between the enduring effects of childhood trauma and the immediate impact of current stressors. Future research should incorporate a broader spectrum of life events, encompassing both distal and proximal factors, to more comprehensively map the multi-temporal pathways to depressive symptoms in both migrant and non-migrant populations. Finally, the measurement of depressive symptoms in this study involves limitations that warrant acknowledgment. We relied exclusively on the CES-D, a self-report screening instrument, rather than clinician-administered diagnostic interviews, which remain the gold standard for psychiatric assessment. While the CES-D is a psychometrically valid tool for identifying individuals at risk, it fundamentally captures subjective distress and is inherently susceptible to interpretive biases. This limitation is particularly salient in light of the Prevalence Inflation Hypothesis (75). As mental health literacy and awareness proliferate, individuals may inadvertently lower their internal threshold for pathologizing normative negative affect—such as transient sadness or situational stress—and report these experiences as psychiatric symptoms. Consequently, the elevated prevalence rates observed in the current study may partially reflect an overestimation where normative emotional fluctuations are conflated with clinical depression. Future research should aim to triangulate these findings using multi-method assessment batteries, including structured clinical interviews, to distinguish between transient psychological distress and formal depressive disorders.

## 5 Conclusions

This study used a large sample of Chinese adolescents and demonstrated that rural-to-urban migration modifies the symptom-level associations between ACEs and depressive symptoms among this population. Distinct bridging symptoms, sexual abuse in rural-to-urban migrants and household substance abuse in non-rural-to-urban migrants, highlight the need for targeted prevention strategies that address the specific psychosocial vulnerabilities of each group.

## Data Availability

The datasets presented in this study can be found in online repositories. The names of the repository/repositories and accession number(s) can be found below: https://osf.io/th4bc/?view_only=1560ff89f1da4c9281c4caa05458a8fd.
