# Supplementary material for: Divergent pathways to depression: a network analysis of adverse childhood experiences in migrant and non-migrant youth
Source: Front Psychiatry. 2026 Feb 27;17:1725710. doi: 10.3389/fpsyt.2026.1725710 (PMC12982459; doi:10.3389/fpsyt.2026.1725710)
Supplement: Supplementary file 1 [file Supplementaryfile1.docx]

Supplementary Material

**Table S1.** Weighted adjacency matrix for non-flow group.

|  | CESD1 | CESD2 | CESD3 | CESD4 | CESD5 | CESD6 | CESD7 | CESD8 | CESD9 | CESD10 | CESD11 | CESD12 | CESD13 | CESD14 | CESD15 | CESD16 | CESD17 | CESD18 | CESD19 | CESD20 | ACE1 | ACE2 | ACE3 | ACE4 | ACE5 | ACE6 | ACE7 | ACE8 | ACE9 | ACE10 | ACE11 | ACE12 |
| --- | --- | --- | --- | --- | --- | --- | --- | --- | --- | --- | --- | --- | --- | --- | --- | --- | --- | --- | --- | --- | --- | --- | --- | --- | --- | --- | --- | --- | --- | --- | --- | --- |
| CESD1 | 0.000 | 0.067 | 0.190 | 0.134 | 0.140 | 0.081 | 0.142 | 0.000 | 0.080 | 0.000 | 0.000 | 0.000 | 0.043 | 0.071 | 0.000 | 0.054 | 0.106 | 0.084 | 0.027 | 0.078 | 0.140 | 0.273 | 0.000 | 0.152 | 0.162 | 0.045 | 0.028 | 0.000 | 0.181 | 0.000 | 0.000 | 0.000 |
| CESD2 | 0.067 | 0.000 | 0.216 | 0.000 | 0.000 | 0.185 | 0.000 | 0.000 | 0.043 | 0.000 | 0.143 | 0.000 | 0.102 | 0.000 | 0.000 | 0.000 | 0.000 | 0.000 | 0.000 | 0.049 | 0.000 | 0.046 | 0.276 | 0.000 | 0.000 | 0.038 | 0.000 | 0.000 | 0.000 | 0.000 | 0.173 | 0.000 |
| CESD3 | 0.190 | 0.216 | 0.000 | 0.000 | 0.078 | 0.100 | 0.047 | 0.000 | 0.028 | 0.083 | 0.000 | 0.000 | 0.000 | 0.033 | 0.038 | 0.000 | 0.050 | 0.092 | 0.000 | 0.095 | 0.050 | 0.082 | 0.000 | 0.104 | 0.000 | 0.044 | 0.123 | 0.038 | 0.000 | 0.094 | 0.000 | 0.034 |
| CESD4 | 0.134 | 0.000 | 0.000 | 0.000 | 0.085 | 0.107 | 0.087 | 0.215 | 0.000 | 0.069 | 0.048 | 0.080 | 0.076 | 0.000 | 0.030 | 0.072 | 0.086 | 0.129 | 0.047 | 0.000 | 0.432 | 0.000 | 0.635 | 0.496 | 0.169 | 0.037 | 0.000 | 0.000 | 0.000 | 0.212 | 0.000 | 0.195 |
| CESD5 | 0.140 | 0.000 | 0.078 | 0.085 | 0.000 | 0.114 | 0.312 | 0.000 | 0.109 | 0.000 | 0.080 | 0.040 | 0.037 | 0.000 | 0.025 | 0.000 | 0.068 | 0.000 | 0.085 | 0.000 | 0.000 | 0.376 | 0.606 | 0.175 | 0.283 | 0.081 | 0.000 | 0.198 | 0.086 | 0.139 | 0.101 | 0.418 |
| CESD6 | 0.081 | 0.185 | 0.100 | 0.107 | 0.114 | 0.000 | 0.221 | 0.075 | 0.073 | 0.047 | 0.080 | 0.000 | 0.126 | 0.032 | 0.000 | 0.000 | 0.000 | 0.187 | 0.035 | 0.125 | 0.000 | 0.053 | 0.271 | 0.114 | 0.111 | 0.170 | 0.000 | 0.000 | 0.000 | 0.000 | 0.000 | 0.033 |
| CESD7 | 0.142 | 0.000 | 0.047 | 0.087 | 0.312 | 0.221 | 0.000 | 0.000 | 0.273 | 0.120 | 0.000 | 0.095 | 0.056 | 0.062 | 0.000 | 0.041 | 0.049 | 0.035 | 0.057 | 0.131 | 0.000 | 0.334 | 0.000 | 0.203 | 0.221 | 0.069 | 0.000 | 0.154 | 0.000 | 0.076 | 0.000 | 0.127 |
| CESD8 | 0.000 | 0.000 | 0.000 | 0.215 | 0.000 | 0.075 | 0.000 | 0.000 | 0.081 | 0.000 | 0.000 | 0.344 | 0.032 | 0.000 | 0.044 | 0.222 | 0.000 | 0.000 | 0.062 | 0.000 | 0.000 | 0.000 | 0.239 | 0.076 | 0.232 | 0.000 | 0.024 | 0.056 | 0.000 | 0.000 | 0.082 | 0.000 |
| CESD9 | 0.080 | 0.043 | 0.028 | 0.000 | 0.109 | 0.073 | 0.273 | 0.081 | 0.000 | 0.192 | 0.101 | 0.079 | 0.000 | 0.000 | 0.206 | 0.106 | 0.000 | 0.046 | 0.000 | 0.087 | 0.000 | 0.466 | 0.272 | 0.189 | 0.000 | 0.037 | 0.000 | 0.211 | 0.089 | 0.000 | 0.000 | 0.000 |
| CESD10 | 0.000 | 0.000 | 0.083 | 0.069 | 0.000 | 0.047 | 0.120 | 0.000 | 0.192 | 0.000 | 0.159 | 0.102 | 0.000 | 0.201 | 0.000 | 0.000 | 0.000 | 0.069 | 0.000 | 0.119 | 0.000 | 0.105 | 0.000 | 0.000 | 0.000 | 0.000 | 0.153 | 0.037 | 0.000 | 0.201 | 0.000 | 0.061 |
| CESD11 | 0.000 | 0.143 | 0.000 | 0.048 | 0.080 | 0.080 | 0.000 | 0.000 | 0.101 | 0.159 | 0.000 | 0.032 | 0.070 | 0.000 | 0.088 | 0.000 | 0.038 | 0.000 | 0.064 | 0.000 | 0.080 | 0.221 | 0.341 | 0.182 | 0.201 | 0.122 | 0.000 | 0.334 | 0.247 | 0.260 | 0.259 | 0.218 |
| CESD12 | 0.000 | 0.000 | 0.000 | 0.080 | 0.040 | 0.000 | 0.095 | 0.344 | 0.079 | 0.102 | 0.032 | 0.000 | 0.079 | 0.000 | 0.000 | 0.475 | 0.026 | 0.085 | 0.000 | 0.000 | 0.038 | 0.176 | 0.254 | 0.211 | 0.000 | 0.000 | 0.071 | 0.138 | 0.084 | 0.048 | 0.000 | 0.080 |
| CESD13 | 0.043 | 0.102 | 0.000 | 0.076 | 0.037 | 0.126 | 0.056 | 0.032 | 0.000 | 0.000 | 0.070 | 0.079 | 0.000 | 0.241 | 0.118 | 0.040 | 0.000 | 0.031 | 0.034 | 0.000 | 0.083 | 0.000 | 0.365 | 0.117 | 0.000 | 0.100 | 0.000 | 0.000 | 0.000 | 0.000 | 0.000 | 0.149 |
| CESD14 | 0.071 | 0.000 | 0.033 | 0.000 | 0.000 | 0.032 | 0.062 | 0.000 | 0.000 | 0.201 | 0.000 | 0.000 | 0.241 | 0.000 | 0.244 | 0.000 | 0.069 | 0.172 | 0.047 | 0.044 | 0.000 | 0.000 | 0.000 | 0.132 | 0.097 | 0.067 | 0.072 | 0.000 | 0.000 | 0.222 | 0.000 | 0.093 |
| CESD15 | 0.000 | 0.000 | 0.038 | 0.030 | 0.025 | 0.000 | 0.000 | 0.044 | 0.206 | 0.000 | 0.088 | 0.000 | 0.118 | 0.244 | 0.000 | 0.065 | 0.089 | 0.170 | 0.348 | 0.126 | 0.081 | 0.038 | 0.494 | 0.331 | 0.217 | 0.128 | 0.000 | 0.185 | 0.000 | 0.120 | 0.100 | 0.365 |
| CESD16 | 0.054 | 0.000 | 0.000 | 0.072 | 0.000 | 0.000 | 0.041 | 0.222 | 0.106 | 0.000 | 0.000 | 0.475 | 0.040 | 0.000 | 0.065 | 0.000 | 0.000 | 0.077 | 0.051 | 0.055 | 0.021 | 0.000 | 0.000 | 0.000 | 0.000 | 0.075 | 0.000 | 0.000 | 0.051 | 0.105 | 0.000 | 0.000 |
| CESD17 | 0.106 | 0.000 | 0.050 | 0.086 | 0.068 | 0.000 | 0.049 | 0.000 | 0.000 | 0.000 | 0.038 | 0.026 | 0.000 | 0.069 | 0.089 | 0.000 | 0.000 | 0.448 | 0.181 | 0.087 | 0.087 | 0.000 | 0.000 | 0.179 | 0.202 | 0.000 | 0.109 | 0.080 | 0.324 | 0.265 | 0.111 | 0.301 |
| CESD18 | 0.084 | 0.000 | 0.092 | 0.129 | 0.000 | 0.187 | 0.035 | 0.000 | 0.046 | 0.069 | 0.000 | 0.085 | 0.031 | 0.172 | 0.170 | 0.077 | 0.448 | 0.000 | 0.221 | 0.133 | 0.068 | 0.149 | 0.000 | 0.049 | 0.360 | 0.074 | 0.176 | 0.000 | 0.283 | 0.246 | 0.000 | 0.000 |
| CESD19 | 0.027 | 0.000 | 0.000 | 0.047 | 0.085 | 0.035 | 0.057 | 0.062 | 0.000 | 0.000 | 0.064 | 0.000 | 0.034 | 0.047 | 0.348 | 0.051 | 0.181 | 0.221 | 0.000 | 0.202 | 0.041 | 0.262 | 0.000 | 0.194 | 0.316 | 0.000 | 0.129 | 0.108 | 0.450 | 0.152 | 0.272 | 0.000 |
| CESD20 | 0.078 | 0.049 | 0.095 | 0.000 | 0.000 | 0.125 | 0.131 | 0.000 | 0.087 | 0.119 | 0.000 | 0.000 | 0.000 | 0.044 | 0.126 | 0.055 | 0.087 | 0.133 | 0.202 | 0.000 | 0.037 | 0.282 | 0.162 | 0.055 | 0.200 | 0.000 | 0.269 | 0.108 | 0.103 | 0.450 | 0.000 | 0.190 |
| ACE1 | 0.140 | 0.000 | 0.050 | 0.432 | 0.000 | 0.000 | 0.000 | 0.000 | 0.000 | 0.000 | 0.080 | 0.038 | 0.083 | 0.000 | 0.081 | 0.021 | 0.087 | 0.068 | 0.041 | 0.037 | 0.000 | 0.696 | 0.390 | 0.278 | 0.059 | 0.088 | 0.160 | 0.390 | 0.000 | 0.466 | 0.000 | 0.000 |
| ACE2 | 0.273 | 0.046 | 0.082 | 0.000 | 0.376 | 0.053 | 0.334 | 0.000 | 0.466 | 0.105 | 0.221 | 0.176 | 0.000 | 0.000 | 0.038 | 0.000 | 0.000 | 0.149 | 0.262 | 0.282 | 0.696 | 0.000 | 1.451 | 0.000 | 0.000 | 0.361 | 0.149 | 0.440 | 0.000 | 0.000 | 0.000 | 0.383 |
| ACE3 | 0.000 | 0.276 | 0.000 | 0.635 | 0.606 | 0.271 | 0.000 | 0.239 | 0.272 | 0.000 | 0.341 | 0.254 | 0.365 | 0.000 | 0.494 | 0.000 | 0.000 | 0.000 | 0.000 | 0.162 | 0.390 | 1.451 | 0.000 | 0.275 | 0.481 | 1.286 | 1.290 | 0.242 | 0.310 | 0.969 | 0.058 | 0.000 |
| ACE4 | 0.152 | 0.000 | 0.104 | 0.496 | 0.175 | 0.114 | 0.203 | 0.076 | 0.189 | 0.000 | 0.182 | 0.211 | 0.117 | 0.132 | 0.331 | 0.000 | 0.179 | 0.049 | 0.194 | 0.055 | 0.278 | 0.000 | 0.275 | 0.000 | 0.865 | 0.000 | 0.291 | 0.159 | 0.183 | 0.412 | 0.209 | 0.341 |
| ACE5 | 0.162 | 0.000 | 0.000 | 0.169 | 0.283 | 0.111 | 0.221 | 0.232 | 0.000 | 0.000 | 0.201 | 0.000 | 0.000 | 0.097 | 0.217 | 0.000 | 0.202 | 0.360 | 0.316 | 0.200 | 0.059 | 0.000 | 0.481 | 0.865 | 0.000 | 0.233 | 0.000 | 0.111 | 0.330 | 1.322 | 0.057 | 0.197 |
| ACE6 | 0.045 | 0.038 | 0.044 | 0.037 | 0.081 | 0.170 | 0.069 | 0.000 | 0.037 | 0.000 | 0.122 | 0.000 | 0.100 | 0.067 | 0.128 | 0.075 | 0.000 | 0.074 | 0.000 | 0.000 | 0.088 | 0.361 | 1.286 | 0.000 | 0.233 | 0.000 | 0.210 | 0.000 | 0.000 | 0.195 | 0.000 | 0.000 |
| ACE7 | 0.028 | 0.000 | 0.123 | 0.000 | 0.000 | 0.000 | 0.000 | 0.024 | 0.000 | 0.153 | 0.000 | 0.071 | 0.000 | 0.072 | 0.000 | 0.000 | 0.109 | 0.176 | 0.129 | 0.269 | 0.160 | 0.149 | 1.290 | 0.291 | 0.000 | 0.210 | 0.000 | 1.057 | 0.508 | 1.251 | 0.355 | 0.239 |
| ACE8 | 0.000 | 0.000 | 0.038 | 0.000 | 0.198 | 0.000 | 0.154 | 0.056 | 0.211 | 0.037 | 0.334 | 0.138 | 0.000 | 0.000 | 0.185 | 0.000 | 0.080 | 0.000 | 0.108 | 0.108 | 0.390 | 0.440 | 0.242 | 0.159 | 0.111 | 0.000 | 1.057 | 0.000 | 1.468 | 0.238 | 0.071 | 0.601 |
| ACE9 | 0.181 | 0.000 | 0.000 | 0.000 | 0.086 | 0.000 | 0.000 | 0.000 | 0.089 | 0.000 | 0.247 | 0.084 | 0.000 | 0.000 | 0.000 | 0.051 | 0.324 | 0.283 | 0.450 | 0.103 | 0.000 | 0.000 | 0.310 | 0.183 | 0.330 | 0.000 | 0.508 | 1.468 | 0.000 | 0.231 | 0.439 | 0.525 |
| ACE10 | 0.000 | 0.000 | 0.094 | 0.212 | 0.139 | 0.000 | 0.076 | 0.000 | 0.000 | 0.201 | 0.260 | 0.048 | 0.000 | 0.222 | 0.120 | 0.105 | 0.265 | 0.246 | 0.152 | 0.450 | 0.466 | 0.000 | 0.969 | 0.412 | 1.322 | 0.195 | 1.251 | 0.238 | 0.231 | 0.000 | 0.000 | 0.445 |
| ACE11 | 0.000 | 0.173 | 0.000 | 0.000 | 0.101 | 0.000 | 0.000 | 0.082 | 0.000 | 0.000 | 0.259 | 0.000 | 0.000 | 0.000 | 0.100 | 0.000 | 0.111 | 0.000 | 0.272 | 0.000 | 0.000 | 0.000 | 0.058 | 0.209 | 0.057 | 0.000 | 0.355 | 0.071 | 0.439 | 0.000 | 0.000 | 0.752 |
| ACE12 | 0.000 | 0.000 | 0.034 | 0.195 | 0.418 | 0.033 | 0.127 | 0.000 | 0.000 | 0.061 | 0.218 | 0.080 | 0.149 | 0.093 | 0.365 | 0.000 | 0.301 | 0.000 | 0.000 | 0.190 | 0.000 | 0.383 | 0.000 | 0.341 | 0.197 | 0.000 | 0.239 | 0.601 | 0.525 | 0.445 | 0.752 | 0.000 |

**Table S2.** Weighted adjacency matrix for flow group.

|  | CESD1 | CESD2 | CESD3 | CESD4 | CESD5 | CESD6 | CESD7 | CESD8 | CESD9 | CESD10 | CESD11 | CESD12 | CESD13 | CESD14 | CESD15 | CESD16 | CESD17 | CESD18 | CESD19 | CESD20 | ACE1 | ACE2 | ACE3 | ACE4 | ACE5 | ACE6 | ACE7 | ACE8 | ACE9 | ACE10 | ACE11 | ACE12 |
| --- | --- | --- | --- | --- | --- | --- | --- | --- | --- | --- | --- | --- | --- | --- | --- | --- | --- | --- | --- | --- | --- | --- | --- | --- | --- | --- | --- | --- | --- | --- | --- | --- |
| CESD1 | 0.000 | 0.053 | 0.143 | 0.000 | 0.115 | 0.089 | 0.000 | 0.000 | 0.000 | 0.000 | 0.000 | 0.000 | 0.000 | 0.000 | 0.000 | 0.000 | 0.059 | 0.127 | 0.000 | 0.000 | 0.000 | 0.000 | 0.000 | 0.000 | 0.000 | 0.000 | 0.000 | 0.000 | 0.000 | 0.108 | 0.000 | 0.000 |
| CESD2 | 0.053 | 0.000 | 0.285 | 0.000 | 0.000 | 0.082 | 0.000 | 0.000 | 0.000 | 0.000 | 0.094 | 0.000 | 0.055 | 0.000 | 0.000 | 0.000 | 0.000 | 0.000 | 0.000 | 0.000 | 0.000 | 0.000 | 0.000 | 0.000 | 0.000 | 0.000 | 0.000 | 0.000 | 0.000 | 0.289 | 0.000 | 0.000 |
| CESD3 | 0.143 | 0.285 | 0.000 | 0.000 | 0.000 | 0.128 | 0.000 | 0.000 | 0.000 | 0.000 | 0.000 | 0.000 | 0.000 | 0.000 | 0.000 | 0.000 | 0.000 | 0.000 | 0.000 | 0.000 | 0.000 | 0.000 | 0.000 | 0.000 | 0.000 | 0.000 | 0.137 | 0.000 | 0.000 | 0.000 | 0.000 | 0.000 |
| CESD4 | 0.000 | 0.000 | 0.000 | 0.000 | 0.000 | 0.000 | 0.000 | 0.193 | 0.000 | 0.000 | 0.000 | 0.155 | 0.000 | 0.000 | 0.000 | 0.180 | 0.000 | 0.000 | 0.000 | 0.000 | 0.273 | 0.000 | 0.000 | 0.000 | 0.000 | 0.000 | 0.000 | 0.000 | 0.000 | 0.106 | 0.000 | 0.000 |
| CESD5 | 0.115 | 0.000 | 0.000 | 0.000 | 0.000 | 0.097 | 0.234 | 0.000 | 0.000 | 0.000 | 0.071 | 0.000 | 0.089 | 0.000 | 0.000 | 0.000 | 0.000 | 0.000 | 0.000 | 0.000 | 0.000 | 0.000 | 0.000 | 0.000 | 0.000 | 0.000 | 0.000 | 0.000 | 0.000 | 0.000 | 0.000 | 0.000 |
| CESD6 | 0.089 | 0.082 | 0.128 | 0.000 | 0.097 | 0.000 | 0.207 | 0.000 | 0.071 | 0.000 | 0.000 | 0.000 | 0.000 | 0.172 | 0.000 | 0.000 | 0.000 | 0.122 | 0.000 | 0.000 | 0.000 | 0.000 | 0.000 | 0.000 | 0.000 | 0.000 | 0.000 | 0.000 | 0.000 | 0.198 | 0.000 | 0.000 |
| CESD7 | 0.000 | 0.000 | 0.000 | 0.000 | 0.234 | 0.207 | 0.000 | 0.000 | 0.179 | 0.112 | 0.000 | 0.000 | 0.000 | 0.000 | 0.081 | 0.000 | 0.000 | 0.000 | 0.085 | 0.000 | 0.000 | 0.000 | 0.000 | 0.000 | 0.000 | 0.000 | 0.000 | 0.000 | 0.000 | 0.237 | 0.000 | 0.000 |
| CESD8 | 0.000 | 0.000 | 0.000 | 0.193 | 0.000 | 0.000 | 0.000 | 0.000 | 0.000 | 0.000 | 0.000 | 0.237 | 0.000 | 0.000 | 0.000 | 0.220 | 0.000 | 0.000 | 0.000 | 0.000 | 0.000 | 0.000 | 0.000 | 0.000 | 0.000 | 0.000 | 0.000 | 0.000 | 0.000 | 0.101 | 0.000 | 0.000 |
| CESD9 | 0.000 | 0.000 | 0.000 | 0.000 | 0.000 | 0.071 | 0.179 | 0.000 | 0.000 | 0.377 | 0.000 | 0.000 | 0.000 | 0.000 | 0.000 | 0.000 | 0.000 | 0.000 | 0.000 | 0.099 | 0.000 | 0.000 | 0.000 | 0.000 | 0.000 | 0.000 | 0.000 | 0.000 | 0.000 | 0.000 | 0.000 | 0.000 |
| CESD10 | 0.000 | 0.000 | 0.000 | 0.000 | 0.000 | 0.000 | 0.112 | 0.000 | 0.377 | 0.000 | 0.097 | 0.000 | 0.000 | 0.000 | 0.084 | 0.000 | 0.084 | 0.062 | 0.000 | 0.000 | 0.000 | 0.000 | 0.000 | 0.000 | 0.000 | 0.000 | 0.000 | 0.000 | 0.000 | 0.000 | 0.000 | 0.000 |
| CESD11 | 0.000 | 0.094 | 0.000 | 0.000 | 0.071 | 0.000 | 0.000 | 0.000 | 0.000 | 0.097 | 0.000 | 0.000 | 0.070 | 0.000 | 0.125 | 0.000 | 0.000 | 0.065 | 0.000 | 0.000 | 0.000 | 0.000 | 0.000 | 0.000 | 0.000 | 0.000 | 0.000 | 0.110 | 0.000 | 0.329 | 0.000 | 0.000 |
| CESD12 | 0.000 | 0.000 | 0.000 | 0.155 | 0.000 | 0.000 | 0.000 | 0.237 | 0.000 | 0.000 | 0.000 | 0.000 | 0.000 | 0.000 | 0.000 | 0.349 | 0.000 | 0.000 | 0.000 | 0.000 | 0.000 | 0.000 | 0.000 | 0.000 | 0.000 | 0.000 | 0.000 | 0.000 | 0.000 | 0.000 | 0.000 | 0.000 |
| CESD13 | 0.000 | 0.055 | 0.000 | 0.000 | 0.089 | 0.000 | 0.000 | 0.000 | 0.000 | 0.000 | 0.070 | 0.000 | 0.000 | 0.191 | 0.000 | 0.000 | 0.000 | 0.000 | 0.000 | 0.000 | 0.000 | 0.000 | 0.000 | 0.000 | 0.000 | 0.000 | 0.000 | 0.000 | 0.000 | 0.149 | 0.000 | 0.000 |
| CESD14 | 0.000 | 0.000 | 0.000 | 0.000 | 0.000 | 0.172 | 0.000 | 0.000 | 0.000 | 0.000 | 0.000 | 0.000 | 0.191 | 0.000 | 0.194 | 0.000 | 0.000 | 0.000 | 0.000 | 0.000 | 0.000 | 0.000 | 0.000 | 0.000 | 0.000 | 0.000 | 0.000 | 0.000 | 0.000 | 0.365 | 0.000 | 0.000 |
| CESD15 | 0.000 | 0.000 | 0.000 | 0.000 | 0.000 | 0.000 | 0.081 | 0.000 | 0.000 | 0.084 | 0.125 | 0.000 | 0.000 | 0.194 | 0.000 | 0.000 | 0.000 | 0.000 | 0.334 | 0.181 | 0.000 | 0.000 | 0.000 | 0.000 | 0.000 | 0.000 | 0.000 | 0.000 | 0.000 | 0.000 | 0.000 | 0.000 |
| CESD16 | 0.000 | 0.000 | 0.000 | 0.180 | 0.000 | 0.000 | 0.000 | 0.220 | 0.000 | 0.000 | 0.000 | 0.349 | 0.000 | 0.000 | 0.000 | 0.000 | 0.000 | 0.000 | 0.000 | 0.000 | 0.000 | 0.000 | 0.000 | 0.000 | 0.000 | 0.000 | 0.000 | 0.000 | 0.000 | 0.000 | 0.000 | 0.000 |
| CESD17 | 0.059 | 0.000 | 0.000 | 0.000 | 0.000 | 0.000 | 0.000 | 0.000 | 0.000 | 0.084 | 0.000 | 0.000 | 0.000 | 0.000 | 0.000 | 0.000 | 0.000 | 0.367 | 0.000 | 0.000 | 0.000 | 0.000 | 0.000 | 0.000 | 0.000 | 0.000 | 0.000 | 0.000 | 0.000 | 0.263 | 0.000 | 0.000 |
| CESD18 | 0.127 | 0.000 | 0.000 | 0.000 | 0.000 | 0.122 | 0.000 | 0.000 | 0.000 | 0.062 | 0.065 | 0.000 | 0.000 | 0.000 | 0.000 | 0.000 | 0.367 | 0.000 | 0.194 | 0.000 | 0.000 | 0.000 | 0.000 | 0.000 | 0.000 | 0.000 | 0.000 | 0.000 | 0.000 | 0.476 | 0.000 | 0.000 |
| CESD19 | 0.000 | 0.000 | 0.000 | 0.000 | 0.000 | 0.000 | 0.085 | 0.000 | 0.000 | 0.000 | 0.000 | 0.000 | 0.000 | 0.000 | 0.334 | 0.000 | 0.000 | 0.194 | 0.000 | 0.300 | 0.000 | 0.000 | 0.000 | 0.000 | 0.000 | 0.000 | 0.000 | 0.000 | 0.000 | 0.000 | 0.000 | 0.000 |
| CESD20 | 0.000 | 0.000 | 0.000 | 0.000 | 0.000 | 0.000 | 0.000 | 0.000 | 0.099 | 0.000 | 0.000 | 0.000 | 0.000 | 0.000 | 0.181 | 0.000 | 0.000 | 0.000 | 0.300 | 0.000 | 0.000 | 0.000 | 0.000 | 0.000 | 0.000 | 0.000 | 0.000 | 0.000 | 0.287 | 0.000 | 0.000 | 0.000 |
| ACE1 | 0.000 | 0.000 | 0.000 | 0.273 | 0.000 | 0.000 | 0.000 | 0.000 | 0.000 | 0.000 | 0.000 | 0.000 | 0.000 | 0.000 | 0.000 | 0.000 | 0.000 | 0.000 | 0.000 | 0.000 | 0.000 | 0.000 | 0.000 | 0.000 | 0.000 | 0.000 | 0.000 | 0.000 | 0.000 | 0.000 | 0.000 | 0.000 |
| ACE2 | 0.000 | 0.000 | 0.000 | 0.000 | 0.000 | 0.000 | 0.000 | 0.000 | 0.000 | 0.000 | 0.000 | 0.000 | 0.000 | 0.000 | 0.000 | 0.000 | 0.000 | 0.000 | 0.000 | 0.000 | 0.000 | 0.000 | 0.364 | 0.339 | 0.000 | 0.000 | 0.000 | 0.266 | 0.524 | 0.000 | 0.000 | 0.571 |
| ACE3 | 0.000 | 0.000 | 0.000 | 0.000 | 0.000 | 0.000 | 0.000 | 0.000 | 0.000 | 0.000 | 0.000 | 0.000 | 0.000 | 0.000 | 0.000 | 0.000 | 0.000 | 0.000 | 0.000 | 0.000 | 0.000 | 0.364 | 0.000 | 0.439 | 0.838 | 0.000 | 0.000 | 0.573 | 0.383 | 0.000 | 0.000 | 0.066 |
| ACE4 | 0.000 | 0.000 | 0.000 | 0.000 | 0.000 | 0.000 | 0.000 | 0.000 | 0.000 | 0.000 | 0.000 | 0.000 | 0.000 | 0.000 | 0.000 | 0.000 | 0.000 | 0.000 | 0.000 | 0.000 | 0.000 | 0.339 | 0.439 | 0.000 | 0.328 | 0.208 | 0.000 | 0.000 | 0.277 | 0.000 | 0.000 | 0.126 |
| ACE5 | 0.000 | 0.000 | 0.000 | 0.000 | 0.000 | 0.000 | 0.000 | 0.000 | 0.000 | 0.000 | 0.000 | 0.000 | 0.000 | 0.000 | 0.000 | 0.000 | 0.000 | 0.000 | 0.000 | 0.000 | 0.000 | 0.000 | 0.838 | 0.328 | 0.000 | 0.284 | 0.000 | 0.000 | 0.000 | 0.000 | 0.000 | 0.286 |
| ACE6 | 0.000 | 0.000 | 0.000 | 0.000 | 0.000 | 0.000 | 0.000 | 0.000 | 0.000 | 0.000 | 0.000 | 0.000 | 0.000 | 0.000 | 0.000 | 0.000 | 0.000 | 0.000 | 0.000 | 0.000 | 0.000 | 0.000 | 0.000 | 0.208 | 0.284 | 0.000 | 0.000 | 0.420 | 0.000 | 0.354 | 0.000 | 0.000 |
| ACE7 | 0.000 | 0.000 | 0.137 | 0.000 | 0.000 | 0.000 | 0.000 | 0.000 | 0.000 | 0.000 | 0.000 | 0.000 | 0.000 | 0.000 | 0.000 | 0.000 | 0.000 | 0.000 | 0.000 | 0.000 | 0.000 | 0.000 | 0.000 | 0.000 | 0.000 | 0.000 | 0.000 | 0.000 | 0.000 | 2.333 | 0.000 | 0.000 |
| ACE8 | 0.000 | 0.000 | 0.000 | 0.000 | 0.000 | 0.000 | 0.000 | 0.000 | 0.000 | 0.000 | 0.110 | 0.000 | 0.000 | 0.000 | 0.000 | 0.000 | 0.000 | 0.000 | 0.000 | 0.000 | 0.000 | 0.266 | 0.573 | 0.000 | 0.000 | 0.420 | 0.000 | 0.000 | 0.671 | 0.000 | 0.552 | 0.379 |
| ACE9 | 0.000 | 0.000 | 0.000 | 0.000 | 0.000 | 0.000 | 0.000 | 0.000 | 0.000 | 0.000 | 0.000 | 0.000 | 0.000 | 0.000 | 0.000 | 0.000 | 0.000 | 0.000 | 0.000 | 0.287 | 0.000 | 0.524 | 0.383 | 0.277 | 0.000 | 0.000 | 0.000 | 0.671 | 0.000 | 0.000 | 0.432 | 0.434 |
| ACE10 | 0.108 | 0.289 | 0.000 | 0.106 | 0.000 | 0.198 | 0.237 | 0.101 | 0.000 | 0.000 | 0.329 | 0.000 | 0.149 | 0.365 | 0.000 | 0.000 | 0.263 | 0.476 | 0.000 | 0.000 | 0.000 | 0.000 | 0.000 | 0.000 | 0.000 | 0.354 | 2.333 | 0.000 | 0.000 | 0.000 | 0.000 | 0.000 |
| ACE11 | 0.000 | 0.000 | 0.000 | 0.000 | 0.000 | 0.000 | 0.000 | 0.000 | 0.000 | 0.000 | 0.000 | 0.000 | 0.000 | 0.000 | 0.000 | 0.000 | 0.000 | 0.000 | 0.000 | 0.000 | 0.000 | 0.000 | 0.000 | 0.000 | 0.000 | 0.000 | 0.000 | 0.552 | 0.432 | 0.000 | 0.000 | 0.392 |
| ACE12 | 0.000 | 0.000 | 0.000 | 0.000 | 0.000 | 0.000 | 0.000 | 0.000 | 0.000 | 0.000 | 0.000 | 0.000 | 0.000 | 0.000 | 0.000 | 0.000 | 0.000 | 0.000 | 0.000 | 0.000 | 0.000 | 0.571 | 0.066 | 0.126 | 0.286 | 0.000 | 0.000 | 0.379 | 0.434 | 0.000 | 0.392 | 0.000 |

**Table S3.** The sensitivity analysis results.

| **Order** | **Original parameter** | **Lower CI** | **Difference** | **Upper CI** |
| --- | --- | --- | --- | --- |
| 1 | 0.05 | -0.05 | 0.00 | 0.07 |
| 2 | 0.16 | -0.07 | 0.00 | 0.07 |
| 3 | 0.22 | -0.09 | -0.01 | 0.07 |
| 4 | -0.11 | -0.05 | 0.01 | 0.07 |
| 5 | 0.00 | 0.00 | 0.00 | 0.06 |
| 6 | 0.01 | -0.01 | -0.01 | 0.05 |
| 7 | 0.13 | -0.08 | 0.00 | 0.08 |
| 8 | 0.00 | -0.05 | 0.00 | 0.04 |
| 9 | 0.06 | -0.06 | 0.00 | 0.07 |
| 10 | -0.11 | -0.06 | 0.00 | 0.07 |
| 11 | 0.08 | -0.08 | 0.00 | 0.07 |
| 12 | 0.18 | -0.08 | 0.00 | 0.08 |
| 13 | 0.09 | -0.08 | 0.00 | 0.09 |
| 14 | 0.07 | -0.07 | -0.01 | 0.05 |
| 15 | 0.10 | -0.10 | -0.01 | 0.07 |
| 16 | 0.15 | -0.06 | 0.00 | 0.07 |
| 17 | 0.00 | 0.00 | 0.00 | 0.06 |
| 18 | 0.05 | -0.05 | -0.01 | 0.07 |
| 19 | -0.04 | -0.05 | 0.02 | 0.04 |
| 20 | 0.21 | -0.09 | -0.01 | 0.07 |
| 21 | 0.16 | -0.08 | 0.00 | 0.08 |
| 22 | 0.00 | -0.04 | 0.00 | 0.03 |
| 23 | -0.02 | -0.05 | 0.01 | 0.02 |
| 24 | 0.00 | -0.02 | 0.00 | 0.04 |
| 25 | 0.20 | -0.08 | -0.01 | 0.07 |
| 26 | 0.00 | -0.06 | 0.00 | 0.03 |
| 27 | -0.03 | -0.05 | 0.01 | 0.03 |
| 28 | 0.00 | -0.05 | 0.00 | 0.01 |
| 29 | 0.06 | -0.06 | 0.00 | 0.06 |
| 30 | 0.06 | -0.06 | -0.01 | 0.06 |
| 31 | 0.03 | -0.03 | 0.00 | 0.07 |
| 32 | 0.00 | -0.01 | 0.00 | 0.02 |
| 33 | -0.05 | -0.07 | 0.02 | 0.05 |
| 34 | 0.05 | -0.05 | 0.00 | 0.07 |
| 35 | 0.29 | -0.08 | -0.01 | 0.07 |
| 36 | 0.00 | 0.00 | 0.00 | 0.07 |
| 37 | 0.00 | 0.00 | 0.00 | 0.05 |
| 38 | 0.03 | -0.03 | -0.01 | 0.07 |
| 39 | 0.10 | -0.08 | -0.01 | 0.08 |
| 40 | 0.04 | -0.04 | -0.01 | 0.05 |
| 41 | 0.01 | -0.01 | -0.01 | 0.07 |
| 42 | 0.04 | -0.04 | 0.00 | 0.09 |
| 43 | 0.11 | -0.09 | -0.01 | 0.08 |
| 44 | 0.00 | -0.05 | 0.00 | 0.00 |
| 45 | 0.16 | -0.08 | 0.00 | 0.08 |
| 46 | 0.00 | -0.02 | 0.00 | 0.07 |
| 47 | 0.10 | -0.08 | 0.00 | 0.07 |
| 48 | 0.02 | -0.02 | 0.00 | 0.08 |
| 49 | -0.04 | -0.06 | 0.01 | 0.04 |
| 50 | 0.12 | -0.08 | 0.00 | 0.07 |
| 51 | 0.08 | -0.08 | -0.01 | 0.06 |
| 52 | 0.00 | 0.00 | 0.00 | 0.07 |
| 53 | 0.00 | -0.04 | 0.00 | 0.05 |
| 54 | 0.05 | -0.05 | -0.01 | 0.06 |
| 55 | 0.11 | -0.09 | -0.01 | 0.06 |
| 56 | 0.00 | -0.01 | 0.00 | 0.06 |
| 57 | 0.00 | -0.04 | 0.00 | 0.01 |
| 58 | 0.00 | -0.02 | 0.00 | 0.03 |
| 59 | 0.06 | -0.06 | 0.00 | 0.07 |
| 60 | -0.02 | -0.06 | 0.01 | 0.03 |
| 61 | 0.00 | -0.03 | 0.00 | 0.01 |
| 62 | 0.06 | -0.06 | -0.01 | 0.06 |
| 63 | 0.35 | -0.09 | 0.00 | 0.08 |
| 64 | 0.08 | -0.08 | -0.01 | 0.06 |
| 65 | -0.05 | -0.04 | 0.01 | 0.05 |
| 66 | 0.03 | -0.04 | -0.01 | 0.05 |
| 67 | -0.01 | -0.06 | 0.01 | 0.01 |
| 68 | 0.10 | -0.08 | -0.01 | 0.06 |
| 69 | 0.00 | 0.00 | 0.00 | 0.06 |
| 70 | 0.08 | -0.07 | -0.01 | 0.06 |
| 71 | 0.00 | -0.02 | 0.00 | 0.06 |
| 72 | 0.11 | -0.07 | 0.00 | 0.07 |
| 73 | 0.00 | -0.05 | 0.00 | 0.00 |
| 74 | -0.05 | -0.06 | 0.01 | 0.05 |
| 75 | 0.00 | 0.00 | 0.00 | 0.04 |
| 76 | 0.00 | 0.00 | 0.00 | 0.07 |
| 77 | 0.04 | -0.04 | 0.00 | 0.08 |
| 78 | -0.07 | -0.06 | 0.00 | 0.07 |
| 79 | 0.04 | -0.04 | 0.00 | 0.07 |
| 80 | 0.02 | -0.02 | -0.01 | 0.07 |
| 81 | 0.06 | -0.06 | -0.01 | 0.08 |
| 82 | 0.03 | -0.03 | 0.00 | 0.06 |
| 83 | 0.00 | 0.00 | 0.00 | 0.07 |
| 84 | 0.04 | -0.04 | 0.00 | 0.09 |
| 85 | 0.06 | -0.06 | -0.01 | 0.07 |
| 86 | 0.00 | -0.05 | 0.00 | 0.00 |
| 87 | 0.03 | -0.03 | 0.00 | 0.08 |
| 88 | 0.22 | -0.09 | -0.01 | 0.08 |
| 89 | 0.00 | -0.02 | 0.00 | 0.06 |
| 90 | 0.00 | 0.00 | 0.00 | 0.04 |
| 91 | 0.22 | -0.09 | -0.01 | 0.07 |
| 92 | 0.00 | -0.05 | 0.00 | 0.02 |
| 93 | 0.00 | 0.00 | 0.00 | 0.07 |
| 94 | 0.02 | -0.02 | -0.01 | 0.05 |
| 95 | -0.04 | -0.06 | 0.00 | 0.04 |
| 96 | 0.05 | -0.05 | 0.00 | 0.08 |
| 97 | 0.00 | 0.00 | 0.00 | 0.05 |
| 98 | 0.00 | -0.01 | 0.00 | 0.05 |
| 99 | -0.01 | -0.07 | 0.01 | 0.02 |
| 100 | 0.15 | -0.08 | -0.01 | 0.06 |
| 101 | 0.01 | -0.01 | -0.01 | 0.08 |
| 102 | 0.13 | -0.08 | 0.00 | 0.08 |
| 103 | 0.00 | -0.02 | 0.00 | 0.07 |
| 104 | 0.09 | -0.09 | -0.01 | 0.06 |
| 105 | 0.18 | -0.10 | -0.01 | 0.07 |
| 106 | 0.06 | -0.06 | -0.01 | 0.04 |
| 107 | 0.00 | -0.03 | 0.00 | 0.02 |
| 108 | 0.00 | -0.01 | 0.00 | 0.04 |
| 109 | 0.05 | -0.05 | -0.01 | 0.07 |
| 110 | 0.00 | -0.03 | 0.00 | 0.06 |
| 111 | 0.00 | 0.00 | 0.00 | 0.05 |
| 112 | 0.03 | -0.03 | 0.00 | 0.07 |
| 113 | 0.22 | -0.08 | 0.00 | 0.08 |
| 114 | 0.10 | -0.08 | -0.01 | 0.06 |
| 115 | 0.00 | -0.04 | 0.00 | 0.00 |
| 116 | 0.02 | -0.02 | -0.01 | 0.06 |
| 117 | 0.48 | -0.08 | 0.00 | 0.08 |
| 118 | -0.04 | -0.06 | 0.00 | 0.04 |
| 119 | 0.00 | 0.00 | 0.00 | 0.03 |
| 120 | 0.05 | -0.05 | -0.01 | 0.06 |
| 121 | 0.08 | -0.08 | 0.00 | 0.08 |
| 122 | 0.00 | -0.07 | 0.00 | 0.02 |
| 123 | -0.01 | -0.06 | 0.01 | 0.01 |
| 124 | -0.08 | -0.07 | 0.00 | 0.06 |
| 125 | 0.09 | -0.08 | 0.00 | 0.07 |
| 126 | 0.01 | -0.01 | 0.00 | 0.08 |
| 127 | -0.04 | -0.06 | 0.01 | 0.04 |
| 128 | 0.02 | -0.03 | 0.00 | 0.06 |
| 129 | 0.00 | -0.03 | 0.00 | 0.03 |
| 130 | 0.00 | -0.03 | 0.00 | 0.05 |
| 131 | 0.06 | -0.06 | 0.00 | 0.08 |
| 132 | 0.01 | -0.03 | -0.01 | 0.04 |
| 133 | -0.01 | -0.06 | 0.01 | 0.03 |
| 134 | -0.01 | -0.07 | 0.01 | 0.01 |
| 135 | 0.06 | -0.06 | 0.00 | 0.08 |
| 136 | 0.00 | -0.03 | 0.00 | 0.05 |
| 137 | 0.04 | -0.04 | -0.01 | 0.06 |
| 138 | 0.00 | 0.00 | 0.00 | 0.05 |
| 139 | 0.10 | -0.08 | 0.00 | 0.07 |
| 140 | 0.12 | -0.06 | 0.00 | 0.06 |
| 141 | 0.00 | 0.00 | 0.00 | 0.07 |
| 142 | 0.16 | -0.08 | -0.01 | 0.07 |
| 143 | 0.00 | -0.03 | 0.00 | 0.00 |
| 144 | 0.00 | -0.03 | 0.00 | 0.03 |
| 145 | 0.00 | -0.03 | 0.00 | 0.00 |
| 146 | 0.09 | -0.07 | -0.01 | 0.07 |
| 147 | 0.00 | -0.02 | 0.00 | 0.05 |
| 148 | -0.07 | -0.07 | 0.00 | 0.06 |
| 149 | 0.04 | -0.04 | 0.00 | 0.07 |
| 150 | 0.15 | -0.08 | 0.00 | 0.08 |
| 151 | -0.11 | -0.06 | 0.02 | 0.10 |
| 152 | -0.08 | -0.06 | 0.00 | 0.06 |
| 153 | 0.30 | -0.10 | -0.01 | 0.08 |
| 154 | -0.03 | -0.06 | 0.01 | 0.03 |
| 155 | 0.00 | -0.04 | 0.00 | 0.02 |
| 156 | 0.00 | 0.00 | 0.00 | 0.07 |
| 157 | 0.03 | -0.03 | -0.01 | 0.06 |
| 158 | 0.09 | -0.08 | -0.01 | 0.06 |
| 159 | 0.01 | -0.01 | -0.01 | 0.06 |
| 160 | -0.03 | -0.06 | 0.01 | 0.03 |
| 161 | 0.04 | -0.04 | 0.00 | 0.06 |
| 162 | 0.00 | -0.04 | 0.00 | 0.02 |
| 163 | 0.01 | -0.01 | -0.01 | 0.06 |
| 164 | 0.07 | -0.07 | 0.00 | 0.08 |
| 165 | 0.00 | -0.04 | 0.00 | 0.04 |
| 166 | 0.04 | -0.04 | 0.00 | 0.07 |
| 167 | 0.02 | -0.02 | -0.01 | 0.08 |
| 168 | 0.37 | -0.10 | -0.01 | 0.08 |
| 169 | -0.03 | -0.06 | 0.00 | 0.03 |
| 170 | 0.20 | -0.08 | 0.00 | 0.07 |
| 171 | 0.18 | -0.09 | -0.01 | 0.06 |
| 172 | 0.00 | -0.04 | 0.00 | 0.00 |
| 173 | 0.02 | -0.02 | -0.01 | 0.07 |
| 174 | 0.08 | -0.07 | 0.00 | 0.08 |
| 175 | 0.02 | -0.02 | -0.01 | 0.05 |
| 176 | 0.00 | -0.05 | 0.00 | 0.00 |
| 177 | 0.00 | -0.05 | 0.00 | 0.00 |
| 178 | 0.09 | -0.08 | 0.00 | 0.06 |
| 179 | 0.00 | -0.04 | 0.00 | 0.00 |
| 180 | 0.08 | -0.07 | 0.00 | 0.08 |
| 181 | 0.12 | -0.08 | 0.00 | 0.08 |
| 182 | -0.01 | -0.06 | 0.01 | 0.01 |
| 183 | 0.00 | -0.01 | 0.00 | 0.02 |
| 184 | 0.00 | 0.00 | 0.00 | 0.05 |
| 185 | 0.07 | -0.07 | 0.00 | 0.09 |
| 186 | 0.05 | -0.05 | -0.01 | 0.09 |
| 187 | -0.02 | -0.04 | 0.01 | 0.02 |
| 188 | -0.04 | -0.06 | 0.01 | 0.04 |
| 189 | 0.08 | -0.07 | 0.00 | 0.07 |
| 190 | 0.12 | -0.09 | -0.01 | 0.07 |
| 191 | -0.06 | -0.06 | 0.00 | 0.06 |
| 192 | 0.00 | -0.06 | 0.00 | 0.01 |
| 193 | 0.01 | -0.01 | -0.01 | 0.05 |
| 194 | 0.20 | -0.07 | 0.00 | 0.06 |
| 195 | 0.00 | -0.05 | 0.00 | 0.04 |
| 196 | 0.00 | -0.02 | 0.00 | 0.04 |
| 197 | 0.00 | -0.05 | 0.00 | 0.01 |
| 198 | 0.01 | -0.04 | -0.01 | 0.07 |
| 199 | 0.00 | -0.03 | 0.00 | 0.03 |
| 200 | 0.00 | -0.02 | 0.00 | 0.04 |
| 201 | 0.03 | -0.03 | 0.00 | 0.07 |
| 202 | 0.03 | -0.04 | -0.01 | 0.05 |
| 203 | -0.02 | -0.05 | 0.01 | 0.02 |
| 204 | 0.00 | -0.03 | 0.00 | 0.02 |
| 205 | 0.04 | -0.04 | -0.01 | 0.06 |
| 206 | 0.01 | -0.03 | 0.00 | 0.06 |
| 207 | 0.02 | -0.03 | 0.00 | 0.06 |
| 208 | 0.00 | -0.05 | 0.00 | 0.01 |
| 209 | 0.03 | -0.03 | 0.00 | 0.07 |
| 210 | 0.00 | -0.01 | 0.00 | 0.06 |
| 211 | 0.08 | -0.06 | 0.00 | 0.06 |
| 212 | 0.00 | 0.00 | 0.00 | 0.04 |
| 213 | 0.00 | -0.03 | 0.00 | 0.00 |
| 214 | 0.00 | -0.05 | 0.00 | 0.00 |
| 215 | -0.15 | -0.06 | 0.00 | 0.07 |
| 216 | 0.00 | -0.02 | 0.00 | 0.00 |
| 217 | 0.14 | -0.07 | -0.01 | 0.05 |
| 218 | 0.00 | -0.04 | 0.00 | 0.01 |
| 219 | 0.22 | -0.06 | 0.01 | 0.07 |
| 220 | 0.00 | -0.04 | 0.00 | 0.00 |
| 221 | -0.07 | -0.06 | 0.00 | 0.07 |
| 222 | 0.08 | -0.07 | -0.01 | 0.05 |
| 223 | 0.00 | -0.03 | 0.00 | 0.00 |
| 224 | 0.00 | 0.00 | 0.00 | 0.00 |
| 225 | 0.00 | -0.06 | 0.00 | 0.00 |
| 226 | 0.00 | 0.00 | 0.00 | 0.07 |
| 227 | -0.01 | -0.06 | -0.01 | 0.01 |
| 228 | -0.05 | -0.03 | 0.02 | 0.05 |
| 229 | -0.13 | -0.05 | 0.00 | 0.07 |
| 230 | 0.06 | -0.06 | 0.00 | 0.06 |
| 231 | -0.10 | -0.05 | 0.00 | 0.06 |
| 232 | 0.00 | 0.00 | 0.00 | 0.01 |
| 233 | 0.13 | -0.06 | -0.02 | 0.03 |
| 234 | 0.00 | 0.00 | 0.00 | 0.04 |
| 235 | 0.16 | -0.06 | -0.01 | 0.04 |
| 236 | -0.15 | -0.05 | 0.00 | 0.06 |
| 237 | 0.05 | -0.04 | 0.01 | 0.06 |
| 238 | 0.00 | 0.00 | 0.00 | 0.02 |
| 239 | -0.07 | -0.03 | 0.01 | 0.06 |
| 240 | 0.08 | -0.06 | -0.01 | 0.04 |
| 241 | 0.00 | 0.00 | 0.00 | 0.05 |
| 242 | -0.07 | -0.05 | 0.01 | 0.06 |
| 243 | -0.06 | -0.04 | 0.01 | 0.06 |
| 244 | 0.11 | -0.06 | 0.00 | 0.05 |
| 245 | 0.00 | 0.00 | 0.00 | 0.04 |
| 246 | -0.12 | -0.05 | 0.01 | 0.07 |
| 247 | -0.03 | -0.05 | -0.01 | 0.03 |
| 248 | -0.06 | -0.05 | 0.01 | 0.06 |
| 249 | 0.02 | -0.02 | -0.01 | 0.05 |
| 250 | 0.00 | -0.06 | 0.00 | 0.00 |
| 251 | 0.07 | -0.07 | -0.01 | 0.06 |
| 252 | -0.01 | -0.05 | 0.00 | 0.01 |
| 253 | 0.28 | -0.07 | 0.00 | 0.07 |
| 254 | -0.04 | -0.05 | 0.01 | 0.04 |
| 255 | 0.00 | -0.02 | 0.00 | 0.03 |
| 256 | -0.01 | -0.05 | 0.01 | 0.01 |
| 257 | -0.09 | -0.06 | 0.00 | 0.07 |
| 258 | 0.01 | -0.02 | 0.00 | 0.07 |
| 259 | 0.00 | -0.03 | 0.00 | 0.03 |
| 260 | -0.01 | -0.05 | 0.00 | 0.01 |
| 261 | 0.00 | -0.02 | 0.00 | 0.05 |
| 262 | -0.01 | -0.05 | 0.01 | 0.01 |
| 263 | 0.00 | -0.03 | 0.00 | 0.02 |
| 264 | 0.03 | -0.04 | 0.00 | 0.06 |
| 265 | 0.02 | -0.02 | -0.01 | 0.04 |
| 266 | -0.01 | -0.04 | 0.01 | 0.01 |
| 267 | -0.03 | -0.05 | 0.01 | 0.03 |
| 268 | 0.07 | -0.07 | -0.01 | 0.07 |
| 269 | 0.00 | -0.03 | 0.00 | 0.03 |
| 270 | 0.04 | -0.04 | 0.00 | 0.06 |
| 271 | 0.00 | -0.06 | 0.00 | 0.00 |
| 272 | 0.04 | -0.04 | 0.00 | 0.07 |
| 273 | 0.00 | -0.05 | 0.00 | 0.00 |
| 274 | 0.01 | -0.03 | -0.01 | 0.07 |
| 275 | 0.01 | -0.02 | -0.01 | 0.07 |
| 276 | 0.02 | -0.05 | -0.02 | 0.11 |
| 277 | -0.04 | -0.05 | 0.00 | 0.04 |
| 278 | 0.00 | -0.03 | 0.00 | 0.01 |
| 279 | 0.00 | -0.04 | 0.00 | 0.01 |
| 280 | -0.03 | -0.06 | 0.00 | 0.03 |
| 281 | 0.04 | -0.04 | -0.01 | 0.05 |
| 282 | -0.02 | -0.04 | 0.01 | 0.02 |
| 283 | -0.02 | -0.04 | 0.00 | 0.02 |
| 284 | 0.04 | -0.04 | -0.01 | 0.04 |
| 285 | 0.00 | -0.04 | 0.00 | 0.02 |
| 286 | 0.00 | -0.04 | 0.00 | 0.00 |
| 287 | 0.03 | -0.03 | 0.00 | 0.06 |
| 288 | 0.00 | -0.02 | 0.00 | 0.04 |
| 289 | 0.00 | -0.04 | 0.00 | 0.02 |
| 290 | -0.03 | -0.05 | 0.01 | 0.03 |
| 291 | 0.04 | -0.04 | 0.00 | 0.06 |
| 292 | 0.00 | -0.02 | 0.00 | 0.04 |
| 293 | 0.04 | -0.04 | -0.01 | 0.05 |
| 294 | -0.07 | -0.05 | 0.02 | 0.07 |
| 295 | 0.06 | -0.06 | -0.01 | 0.06 |
| 296 | -0.03 | -0.04 | 0.02 | 0.03 |
| 297 | 0.00 | -0.05 | 0.00 | 0.04 |
| 298 | 0.00 | -0.04 | 0.00 | 0.05 |
| 299 | 0.03 | -0.05 | -0.01 | 0.11 |
| 300 | 0.28 | -0.18 | 0.00 | 0.17 |
| 301 | 0.00 | -0.03 | 0.00 | 0.02 |
| 302 | 0.02 | -0.02 | -0.01 | 0.07 |
| 303 | 0.00 | 0.00 | 0.00 | 0.06 |
| 304 | 0.02 | -0.02 | 0.00 | 0.07 |
| 305 | -0.04 | -0.07 | 0.00 | 0.04 |
| 306 | 0.05 | -0.05 | -0.01 | 0.06 |
| 307 | 0.00 | -0.02 | 0.00 | 0.00 |
| 308 | -0.01 | -0.05 | 0.01 | 0.01 |
| 309 | 0.00 | 0.00 | 0.00 | 0.04 |
| 310 | 0.00 | 0.00 | 0.00 | 0.03 |
| 311 | -0.04 | -0.07 | 0.00 | 0.04 |
| 312 | 0.00 | -0.05 | 0.00 | 0.00 |
| 313 | 0.05 | -0.05 | 0.00 | 0.07 |
| 314 | 0.02 | -0.02 | -0.01 | 0.06 |
| 315 | -0.04 | -0.07 | 0.00 | 0.04 |
| 316 | -0.04 | -0.04 | 0.01 | 0.04 |
| 317 | 0.00 | -0.05 | 0.00 | 0.02 |
| 318 | 0.04 | -0.04 | -0.01 | 0.05 |
| 319 | 0.00 | -0.04 | 0.00 | 0.02 |
| 320 | 0.00 | 0.00 | 0.00 | 0.05 |
| 321 | -0.01 | -0.06 | 0.00 | 0.02 |
| 322 | 0.06 | -0.06 | 0.00 | 0.06 |
| 323 | 0.28 | -0.07 | -0.01 | 0.06 |
| 324 | 0.00 | -0.02 | 0.00 | 0.07 |
| 325 | 0.03 | -0.04 | -0.02 | 0.06 |
| 326 | 0.00 | 0.00 | 0.00 | 0.03 |
| 327 | 0.00 | 0.00 | 0.00 | 0.05 |
| 328 | 0.05 | -0.05 | -0.01 | 0.06 |
| 329 | 0.00 | 0.00 | 0.00 | 0.07 |
| 330 | 0.00 | -0.05 | 0.00 | 0.01 |
| 331 | 0.00 | -0.01 | 0.00 | 0.00 |
| 332 | 0.00 | 0.00 | 0.00 | 0.05 |
| 333 | -0.01 | -0.05 | 0.01 | 0.01 |
| 334 | 0.00 | 0.00 | 0.00 | 0.04 |
| 335 | 0.06 | -0.06 | 0.00 | 0.07 |
| 336 | -0.02 | -0.07 | 0.00 | 0.02 |
| 337 | -0.01 | -0.05 | 0.00 | 0.01 |
| 338 | 0.00 | 0.00 | 0.00 | 0.04 |
| 339 | 0.03 | -0.03 | 0.00 | 0.07 |
| 340 | 0.00 | -0.07 | 0.00 | 0.00 |
| 341 | -0.03 | -0.05 | 0.00 | 0.03 |
| 342 | -0.04 | -0.06 | 0.00 | 0.04 |
| 343 | 0.06 | -0.06 | 0.00 | 0.07 |
| 344 | -0.03 | -0.06 | 0.02 | 0.03 |
| 345 | 0.14 | -0.08 | 0.00 | 0.09 |
| 346 | 0.00 | -0.02 | 0.00 | 0.07 |
| 347 | 0.02 | -0.02 | 0.00 | 0.06 |
| 348 | 0.19 | -0.08 | -0.01 | 0.07 |
| 349 | 0.01 | -0.01 | -0.01 | 0.08 |
| 350 | 0.00 | -0.07 | 0.00 | 0.02 |
| 351 | 0.04 | -0.04 | -0.01 | 0.07 |
| 352 | 0.00 | -0.04 | 0.00 | 0.00 |
| 353 | 0.00 | -0.03 | 0.00 | 0.01 |
| 354 | 0.00 | -0.03 | 0.00 | 0.02 |
| 355 | 0.00 | -0.05 | 0.00 | 0.01 |
| 356 | 0.02 | -0.02 | -0.01 | 0.05 |
| 357 | 0.00 | -0.03 | 0.00 | 0.00 |
| 358 | -0.03 | -0.04 | 0.01 | 0.03 |
| 359 | 0.00 | -0.02 | 0.00 | 0.04 |
| 360 | -0.03 | -0.03 | 0.02 | 0.03 |
| 361 | -0.01 | -0.04 | 0.01 | 0.01 |
| 362 | 0.06 | -0.06 | 0.00 | 0.06 |
| 363 | 0.02 | -0.02 | -0.01 | 0.04 |
| 364 | 0.00 | -0.03 | 0.00 | 0.02 |
| 365 | -0.02 | -0.04 | 0.01 | 0.02 |
| 366 | 0.04 | -0.04 | -0.01 | 0.06 |
| 367 | 0.00 | -0.02 | 0.00 | 0.03 |
| 368 | 0.02 | -0.02 | 0.00 | 0.06 |
| 369 | 0.00 | -0.05 | 0.00 | 0.00 |
| 370 | 0.02 | -0.02 | 0.00 | 0.07 |
| 371 | -0.01 | -0.05 | 0.01 | 0.01 |
| 372 | 0.03 | -0.04 | -0.01 | 0.06 |
| 373 | 0.04 | -0.04 | -0.01 | 0.06 |
| 374 | -0.03 | -0.07 | 0.02 | 0.04 |
| 375 | 0.06 | -0.15 | -0.01 | 0.15 |
| 376 | 0.02 | -0.12 | 0.00 | 0.17 |
| 377 | 0.00 | -0.03 | 0.00 | 0.06 |
| 378 | 0.08 | -0.08 | -0.01 | 0.08 |
| 379 | -0.04 | -0.03 | 0.01 | 0.04 |
| 380 | 0.00 | -0.01 | 0.00 | 0.01 |
| 381 | -0.01 | -0.03 | 0.01 | 0.01 |
| 382 | -0.04 | -0.04 | 0.01 | 0.04 |
| 383 | 0.01 | -0.02 | -0.01 | 0.05 |
| 384 | -0.01 | -0.03 | 0.01 | 0.01 |
| 385 | 0.00 | -0.02 | 0.00 | 0.00 |
| 386 | 0.00 | -0.02 | 0.00 | 0.02 |
| 387 | 0.00 | -0.05 | 0.00 | 0.00 |
| 388 | 0.00 | -0.02 | 0.00 | 0.02 |
| 389 | 0.04 | -0.04 | -0.01 | 0.06 |
| 390 | 0.00 | -0.03 | 0.00 | 0.01 |
| 391 | 0.00 | -0.03 | 0.00 | 0.00 |
| 392 | -0.01 | -0.04 | 0.00 | 0.01 |
| 393 | 0.00 | 0.00 | 0.01 | 0.07 |
| 394 | -0.01 | -0.04 | 0.01 | 0.01 |
| 395 | 0.05 | -0.05 | -0.01 | 0.05 |
| 396 | -0.05 | -0.04 | 0.02 | 0.05 |
| 397 | 0.08 | -0.08 | -0.02 | 0.05 |
| 398 | 0.00 | -0.01 | 0.00 | 0.04 |
| 399 | 0.00 | -0.03 | 0.00 | 0.06 |
| 400 | 0.05 | -0.05 | -0.01 | 0.07 |
| 401 | -0.01 | -0.10 | 0.01 | 0.01 |
| 402 | 0.09 | -0.13 | 0.00 | 0.19 |
| 403 | 0.11 | -0.15 | -0.01 | 0.17 |
| 404 | 0.01 | -0.02 | -0.01 | 0.06 |
| 405 | 0.04 | -0.04 | -0.01 | 0.07 |
| 406 | 0.47 | -0.21 | 0.00 | 0.18 |
| 407 | 0.00 | 0.00 | 0.00 | 0.00 |
| 408 | 0.00 | 0.00 | 0.00 | 0.05 |
| 409 | 0.02 | -0.02 | 0.00 | 0.06 |
| 410 | 0.04 | -0.04 | 0.00 | 0.05 |
| 411 | -0.02 | -0.06 | 0.00 | 0.02 |
| 412 | 0.00 | -0.02 | 0.00 | 0.00 |
| 413 | 0.00 | 0.00 | 0.00 | 0.05 |
| 414 | 0.00 | -0.04 | 0.00 | 0.00 |
| 415 | 0.00 | 0.00 | 0.00 | 0.02 |
| 416 | 0.06 | -0.06 | 0.00 | 0.07 |
| 417 | -0.07 | -0.06 | 0.01 | 0.07 |
| 418 | 0.00 | -0.03 | 0.00 | 0.00 |
| 419 | 0.00 | 0.00 | 0.00 | 0.04 |
| 420 | 0.07 | -0.07 | -0.01 | 0.07 |
| 421 | -0.03 | -0.07 | 0.01 | 0.03 |
| 422 | -0.03 | -0.04 | 0.01 | 0.03 |
| 423 | -0.08 | -0.06 | 0.01 | 0.08 |
| 424 | 0.07 | -0.07 | 0.00 | 0.07 |
| 425 | -0.02 | -0.06 | 0.01 | 0.02 |
| 426 | 0.19 | -0.09 | -0.01 | 0.08 |
| 427 | -0.05 | -0.06 | 0.00 | 0.05 |
| 428 | 0.02 | -0.02 | 0.00 | 0.06 |
| 429 | 0.20 | -0.07 | 0.00 | 0.08 |
| 430 | 0.05 | -0.05 | -0.02 | 0.07 |
| 431 | 0.10 | -0.10 | -0.01 | 0.10 |
| 432 | 0.05 | -0.05 | 0.00 | 0.07 |
| 433 | 0.38 | -0.10 | -0.01 | 0.09 |
| 434 | 0.00 | -0.06 | 0.00 | 0.03 |
| 435 | 0.02 | -0.02 | 0.00 | 0.07 |
| 436 | 0.00 | -0.04 | 0.00 | 0.01 |
| 437 | -0.02 | -0.04 | 0.01 | 0.02 |
| 438 | -0.01 | -0.04 | 0.01 | 0.01 |
| 439 | 0.00 | -0.05 | 0.00 | 0.03 |
| 440 | 0.01 | -0.03 | 0.00 | 0.05 |
| 441 | 0.00 | -0.02 | 0.00 | 0.01 |
| 442 | 0.00 | -0.02 | 0.00 | 0.03 |
| 443 | 0.00 | -0.01 | 0.00 | 0.04 |
| 444 | 0.00 | -0.03 | 0.00 | 0.00 |
| 445 | -0.01 | -0.04 | 0.01 | 0.01 |
| 446 | 0.03 | -0.03 | -0.01 | 0.05 |
| 447 | 0.00 | -0.04 | 0.00 | 0.02 |
| 448 | 0.00 | -0.02 | 0.00 | 0.01 |
| 449 | 0.00 | -0.03 | 0.00 | 0.01 |
| 450 | 0.02 | -0.02 | 0.00 | 0.06 |
| 451 | 0.00 | -0.03 | 0.00 | 0.02 |
| 452 | 0.01 | -0.02 | 0.00 | 0.06 |
| 453 | 0.00 | -0.04 | 0.00 | 0.00 |
| 454 | 0.04 | -0.04 | -0.01 | 0.06 |
| 455 | 0.00 | -0.04 | 0.00 | 0.00 |
| 456 | -0.01 | -0.04 | 0.01 | 0.03 |
| 457 | 0.02 | -0.02 | 0.00 | 0.08 |
| 458 | 0.00 | -0.10 | 0.00 | 0.05 |
| 459 | 0.08 | -0.15 | -0.01 | 0.17 |
| 460 | 0.03 | -0.14 | -0.01 | 0.18 |
| 461 | 0.00 | -0.05 | 0.00 | 0.06 |
| 462 | 0.00 | 0.00 | 0.00 | 0.07 |
| 463 | 0.02 | -0.16 | 0.00 | 0.19 |
| 464 | 0.18 | -0.18 | -0.01 | 0.19 |
| 465 | 0.00 | 0.00 | 0.00 | 0.07 |
| 466 | -0.01 | -0.05 | 0.01 | 0.02 |
| 467 | 0.00 | -0.02 | 0.00 | 0.03 |
| 468 | -0.01 | -0.04 | 0.01 | 0.01 |
| 469 | -0.05 | -0.06 | 0.00 | 0.05 |
| 470 | 0.07 | -0.07 | -0.01 | 0.06 |
| 471 | -0.01 | -0.05 | 0.01 | 0.01 |
| 472 | -0.02 | -0.04 | 0.01 | 0.02 |
| 473 | 0.00 | -0.04 | 0.00 | 0.03 |
| 474 | -0.01 | -0.04 | 0.01 | 0.01 |
| 475 | -0.02 | -0.05 | 0.01 | 0.02 |
| 476 | 0.03 | -0.05 | 0.00 | 0.08 |
| 477 | -0.02 | -0.03 | 0.01 | 0.02 |
| 478 | -0.01 | -0.04 | 0.01 | 0.01 |
| 479 | -0.03 | -0.05 | 0.01 | 0.03 |
| 480 | 0.08 | -0.08 | -0.01 | 0.07 |
| 481 | 0.00 | -0.04 | 0.00 | 0.02 |
| 482 | 0.06 | -0.06 | 0.00 | 0.06 |
| 483 | -0.01 | -0.06 | 0.00 | 0.01 |
| 484 | 0.00 | -0.01 | 0.00 | 0.07 |
| 485 | -0.02 | -0.06 | 0.01 | 0.02 |
| 486 | 0.00 | -0.06 | 0.00 | 0.04 |
| 487 | 0.07 | -0.07 | -0.01 | 0.06 |
| 488 | 0.00 | -0.05 | 0.00 | 0.06 |
| 489 | 0.13 | -0.13 | 0.00 | 0.17 |
| 490 | 0.09 | -0.13 | 0.00 | 0.15 |
| 491 | 0.01 | -0.03 | -0.01 | 0.05 |
| 492 | 0.00 | 0.00 | 0.00 | 0.06 |
| 493 | 0.15 | -0.15 | -0.01 | 0.18 |
| 494 | 0.19 | -0.19 | 0.00 | 0.18 |
| 495 | 0.05 | -0.05 | -0.02 | 0.06 |
| 496 | 0.21 | -0.19 | -0.02 | 0.16 |

**
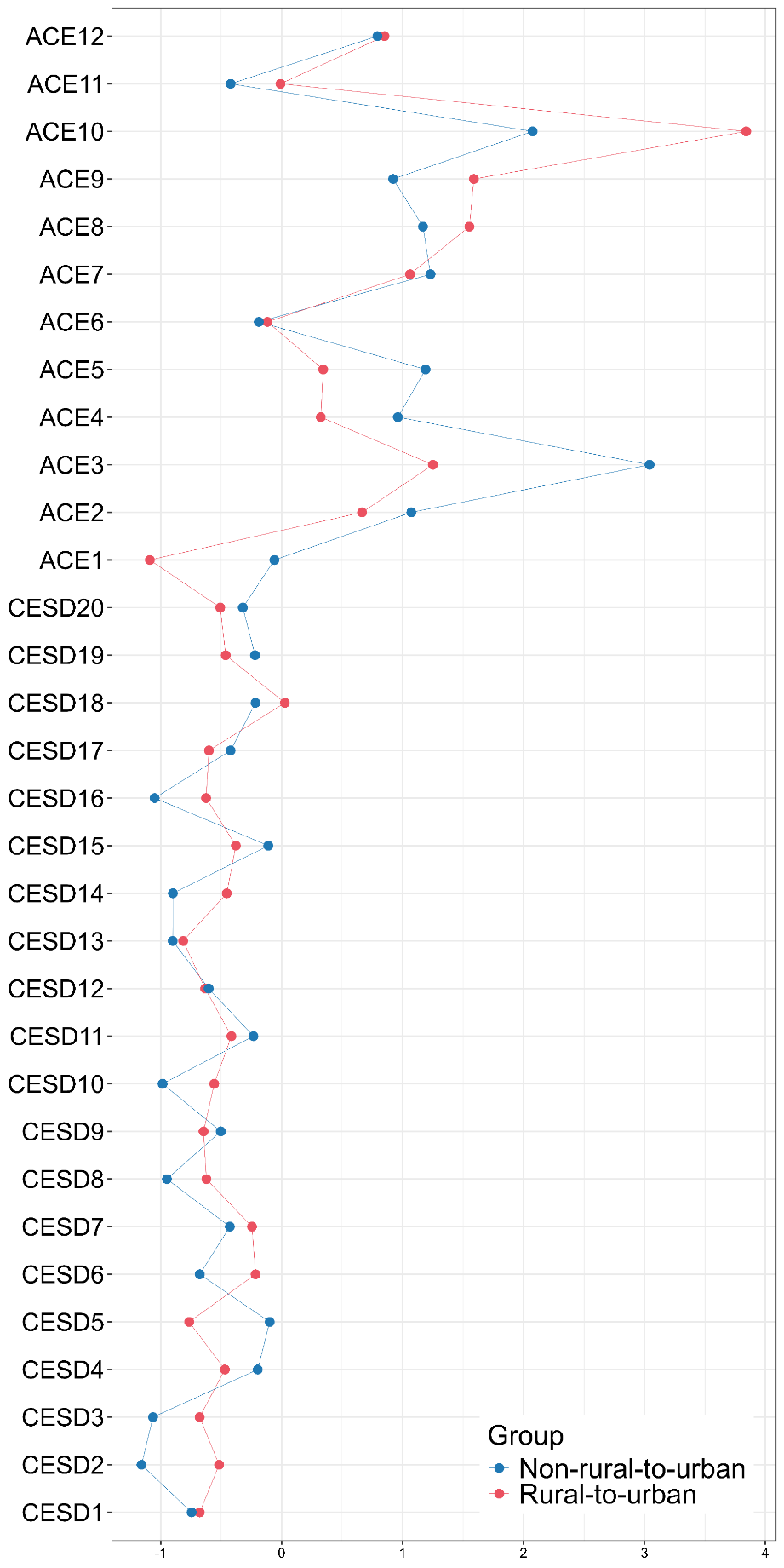
**

**Figure S1.** The EI values for all nodes.

**
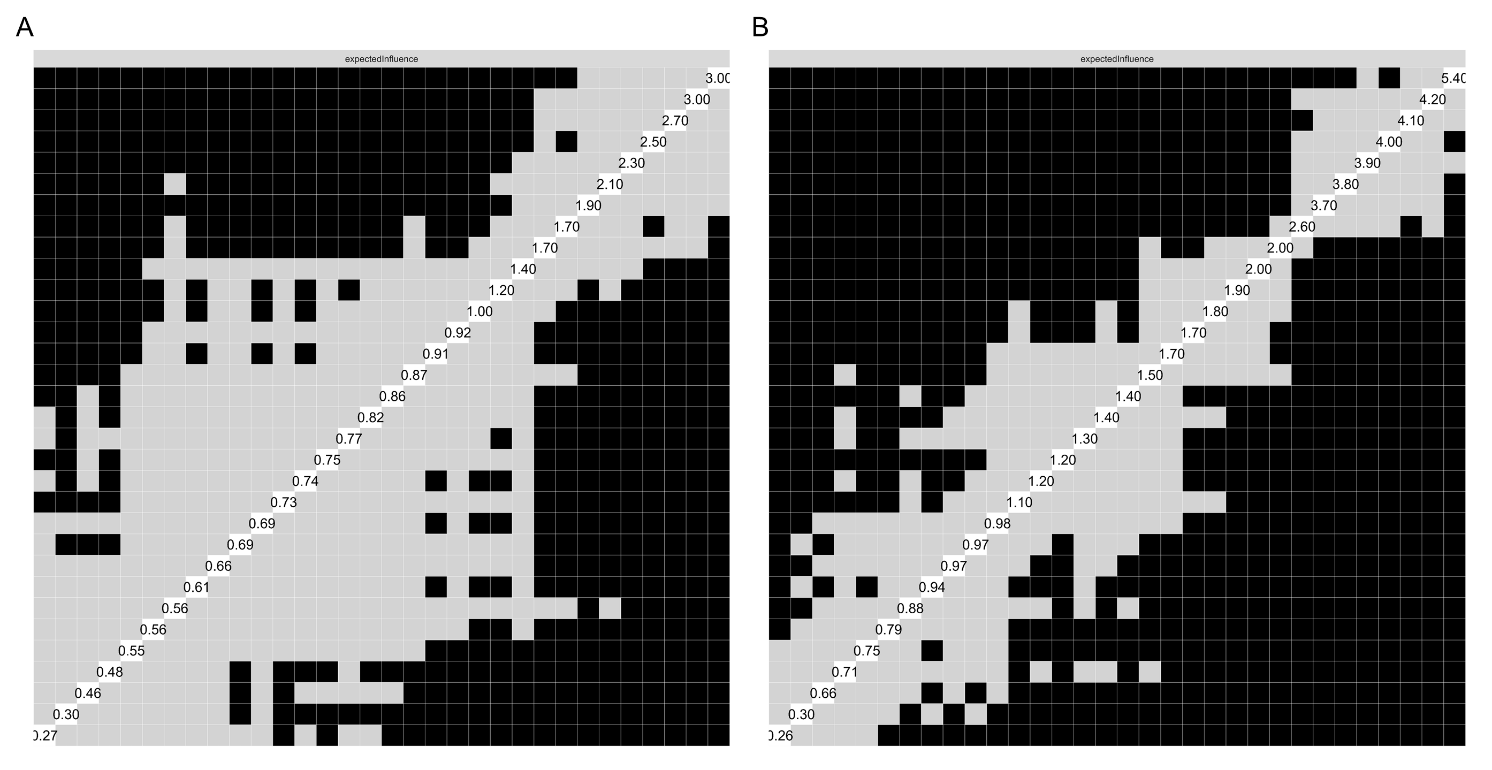
**

**Figure S2.** Nonparametric bootstrapped difference test for each node’s EI value. Grey boxes indicate no significant difference, whereas black boxes indicate a statistically significant difference (*p* < 0.05). A, migration group. B, non-migration group.

**
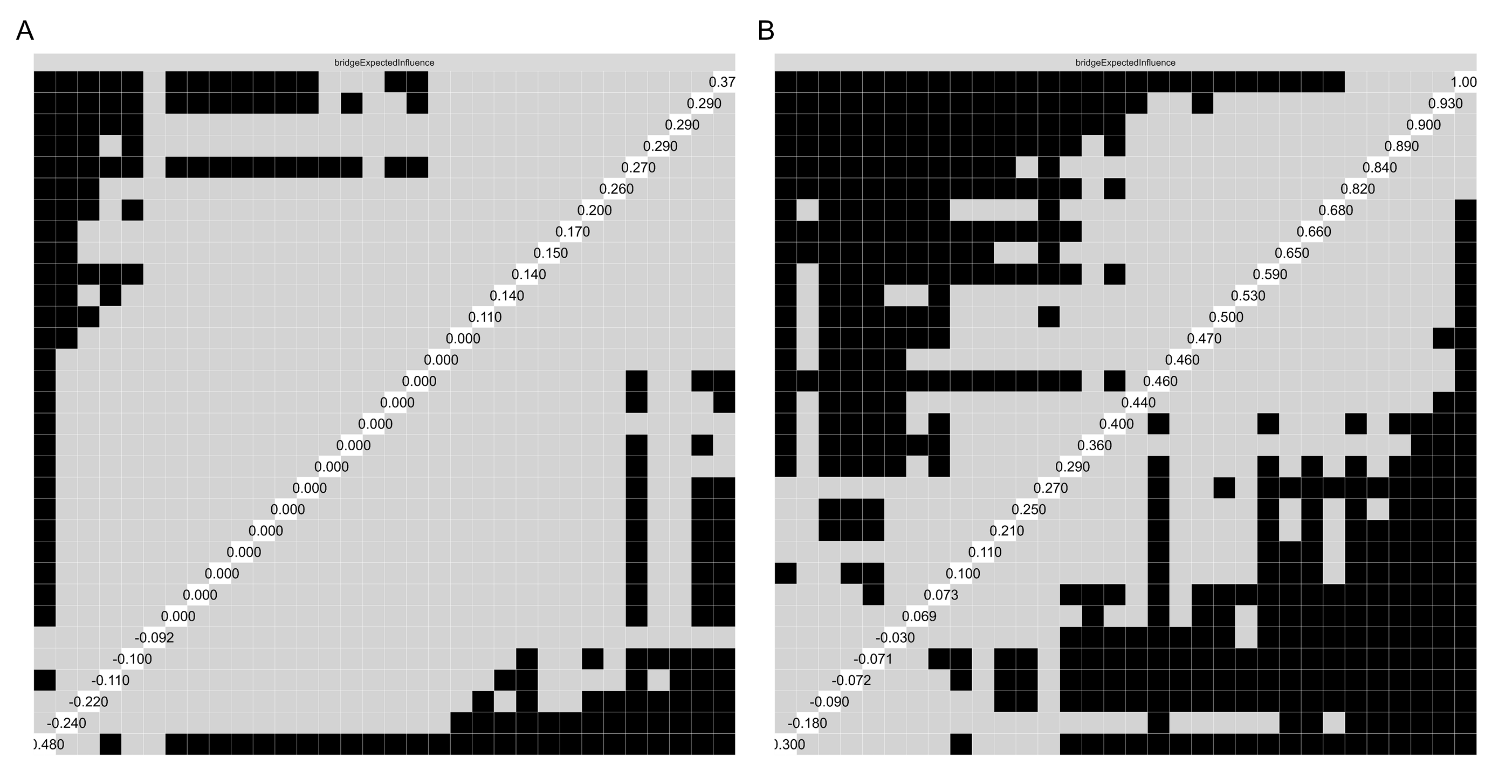
**

**Figure S3.** The results of the bootstrapped difference tests (*α* = 0.05) for bridge EIs are shown in this figure. The color of the boxes indicates whether edge-weights differ significantly from each other (i.e., black) or do not differ significantly (i.e., grey). A, migration group. B, non-migration group.

**
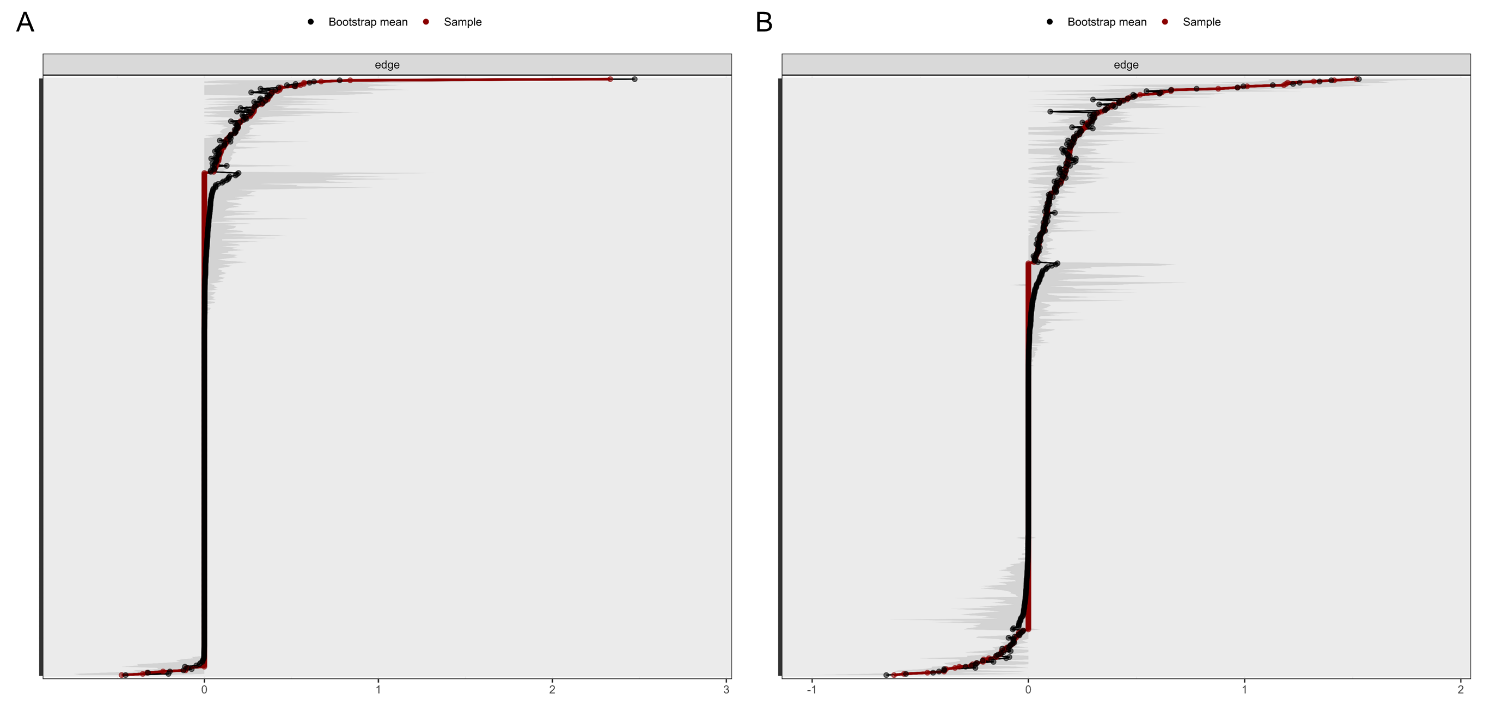
**

**Figure S4.** Nonparametric bootstrapped confidence intervals of estimated edges. The red line represents the estimated edge, while the dark area indicates the 95% bootstrap confidence interval. A, migration group. B, non-migration group.

**
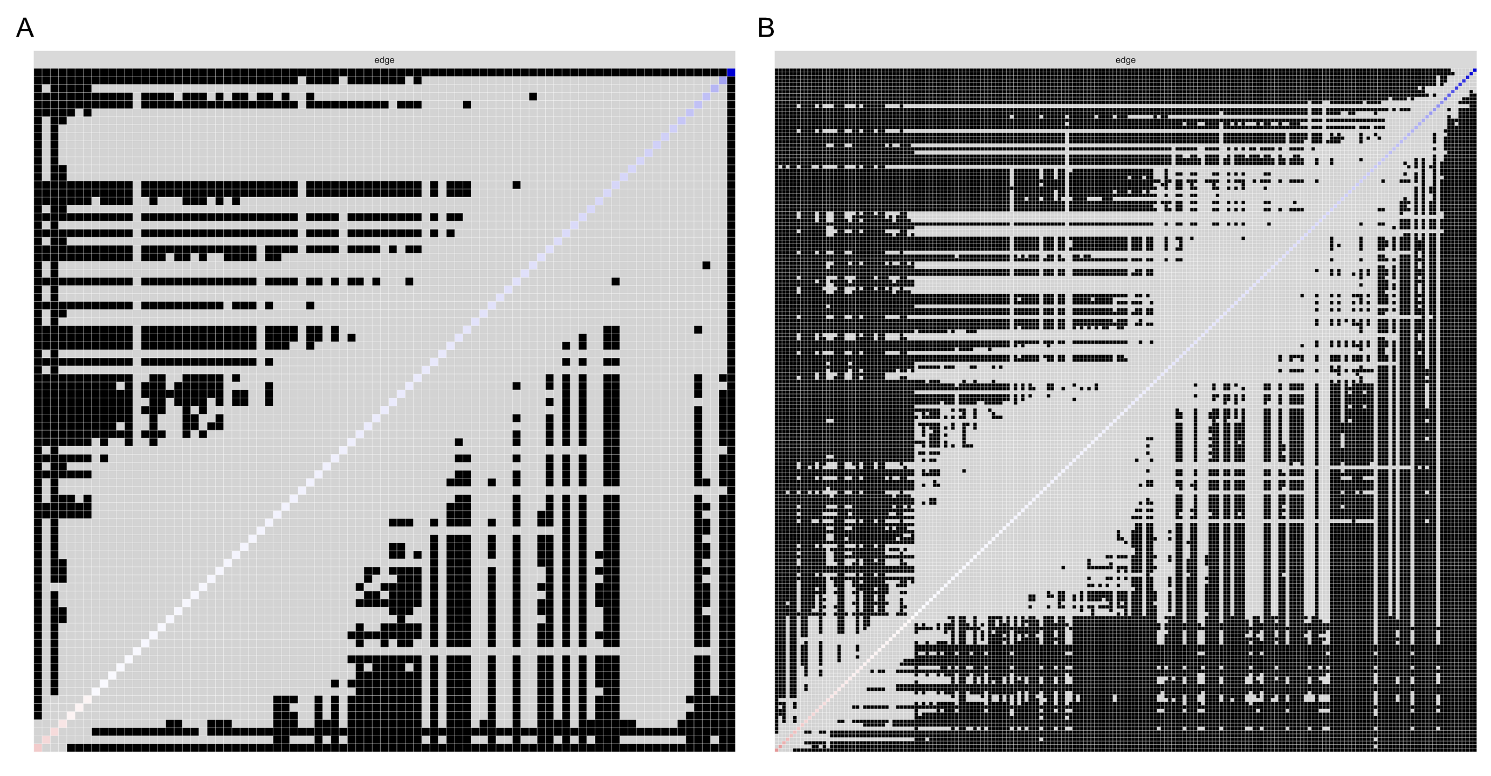
**

**Figure S5.** The results of the bootstrapped difference tests (*α* = 0.05) for pairwise edge weights are shown in this figure. The color of the boxes indicates whether edge-weights differ significantly from each other (i.e., black) or do not differ significantly (i.e., grey). A, migration group. B, non-migration group.

**
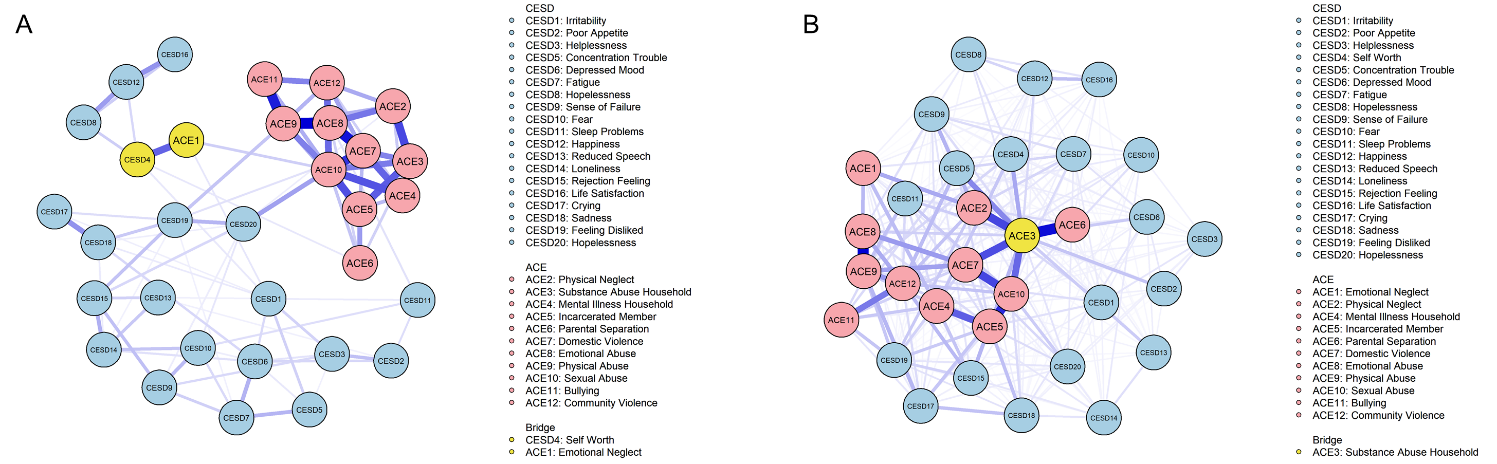
**

**Figure S6.** The depression-ACE network structures of urban group (Part A) and rural group (Part B).

**
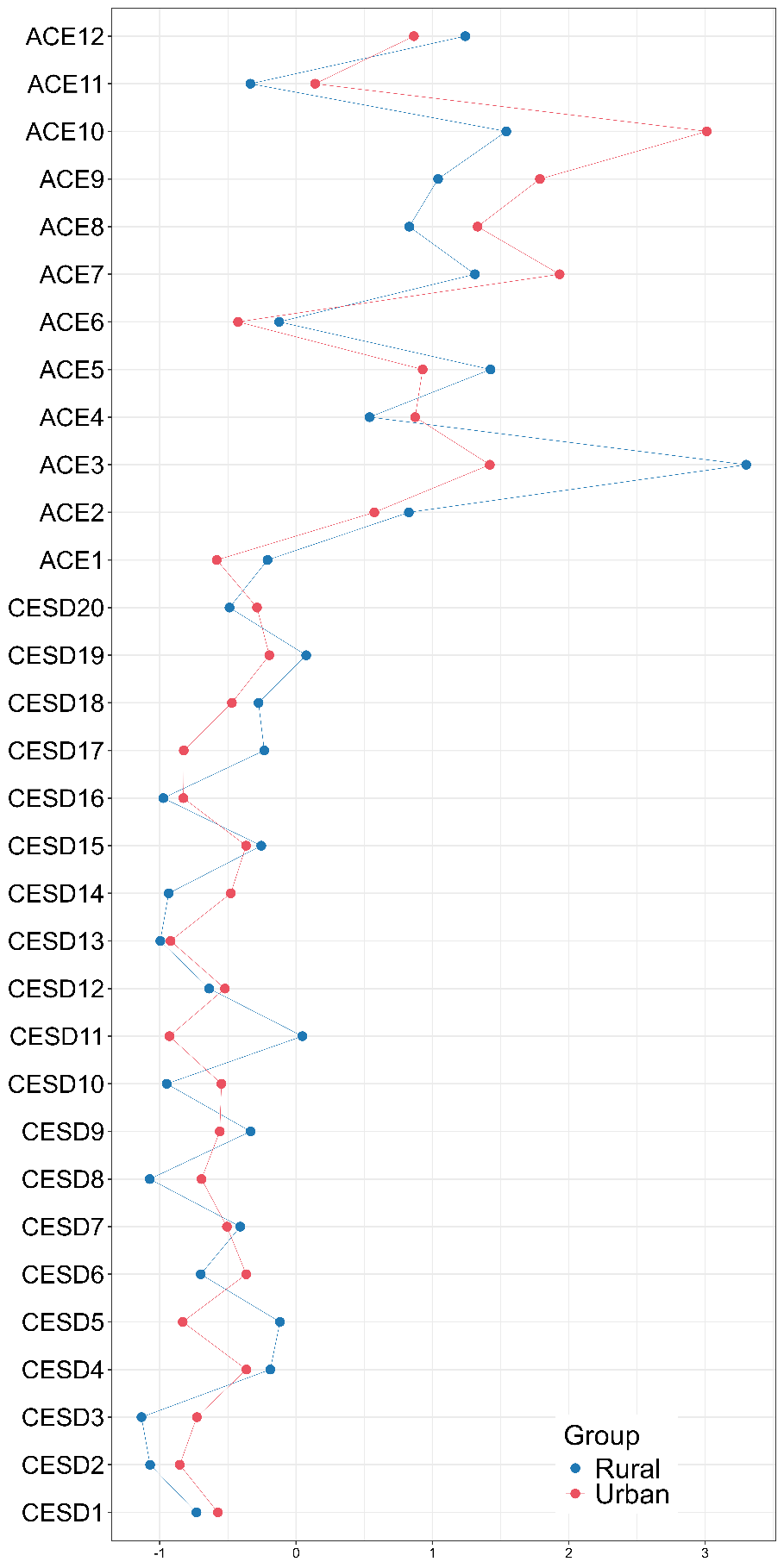
**

**Figure S7.** The EI values of urban group and rural group.

**
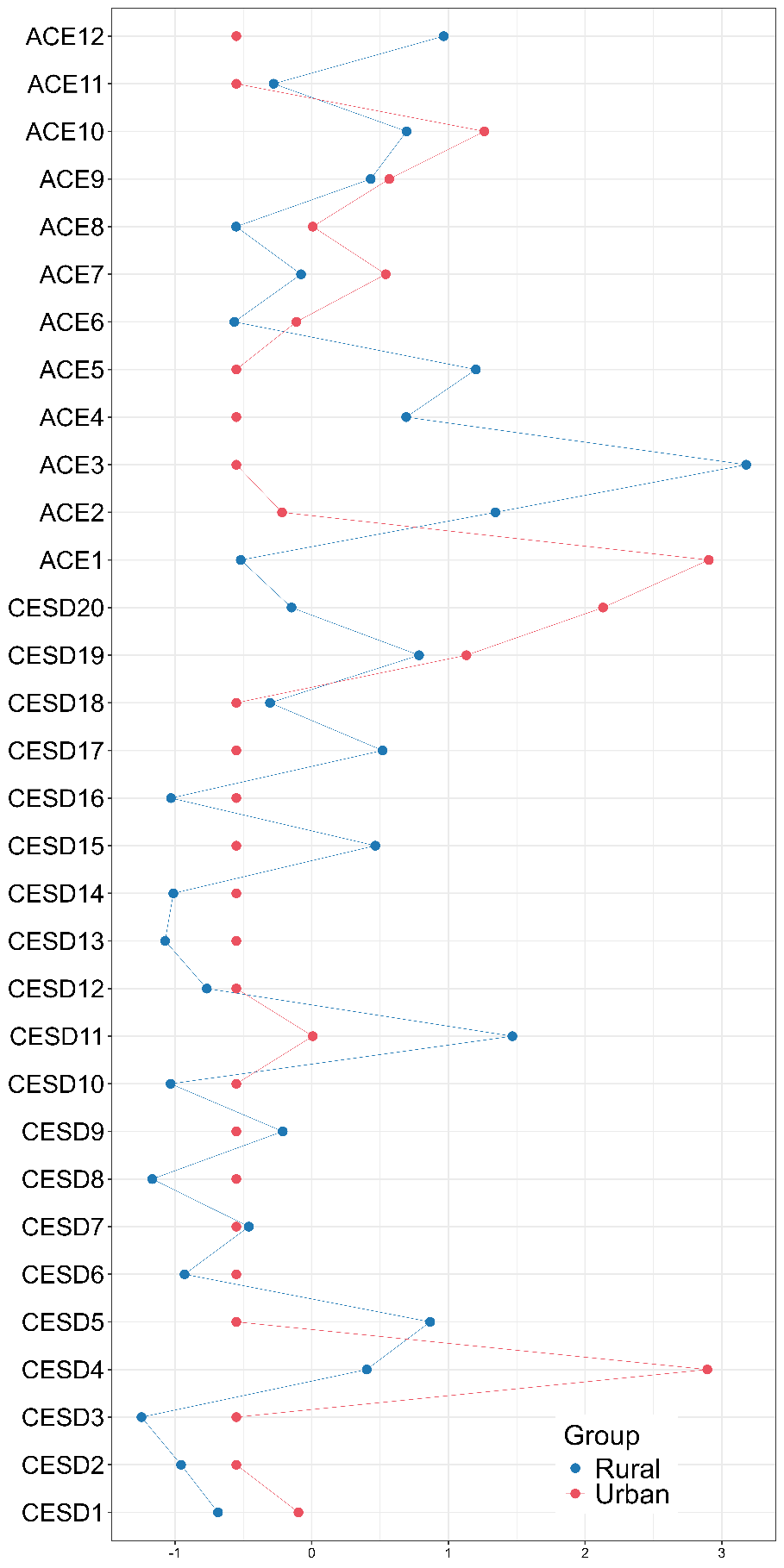
**

**Figure S8.** The bridge EI values of urban group and rural group.

**
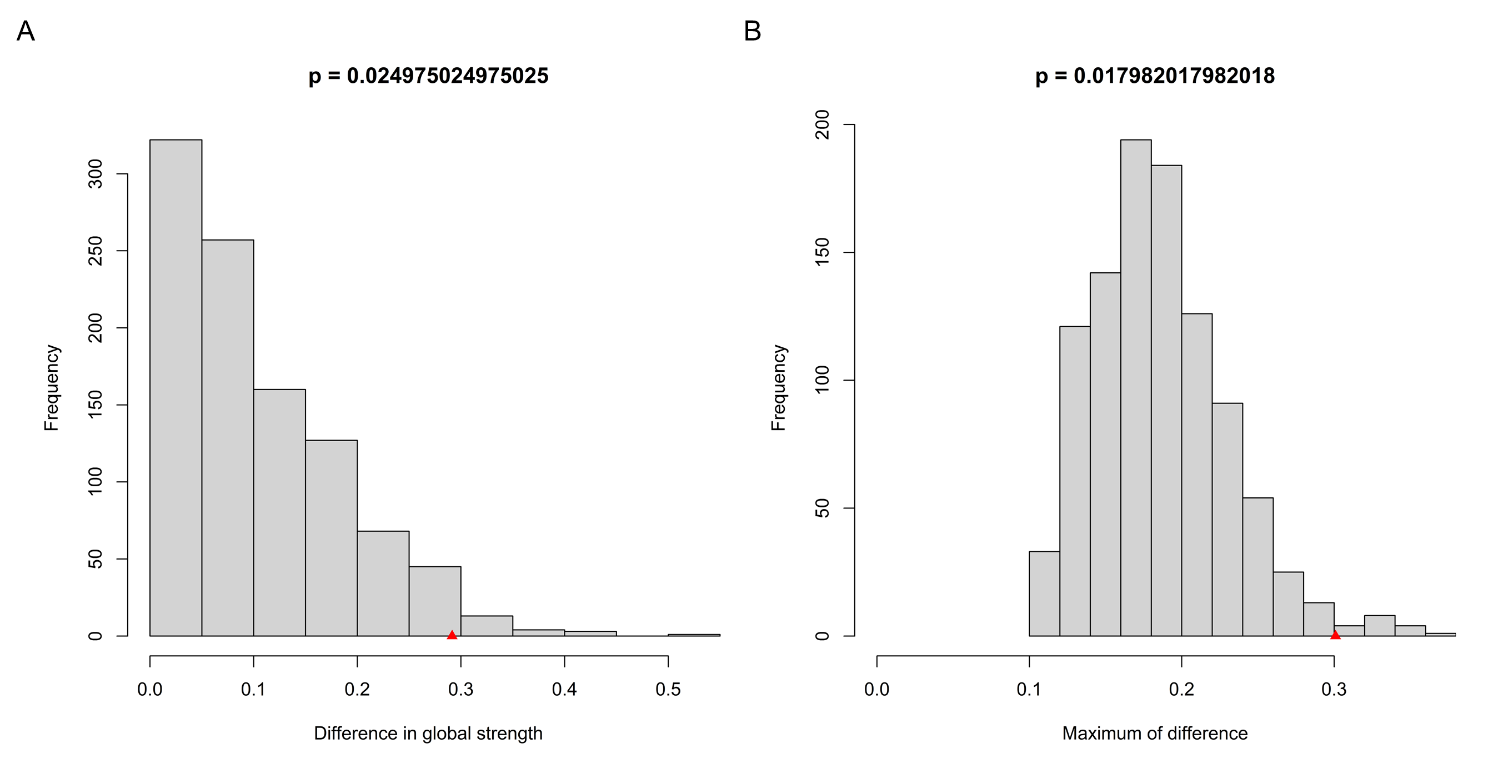
**

**Figure S9.** Network comparison test results between urban and rural groups. A, network global invariance test. B, network structure invariance test.
